# Mapping the Carbon Footprint of Organic Chemicals in Commerce

**DOI:** 10.1021/acs.est.6c06036

**Published:** 2026-10-28

**Authors:** Dachuan Zhang, Zhanyun Wang, Christopher Oberschelp, Jing Huo, Stefanie Hellweg

**Affiliations:** † Ecological Systems Design, 27219 Institute of Environmental Engineering, ETH Zürich, Zürich 8093, Switzerland; ‡ National Centre of Competence in Research (NCCR) Catalysis, Zürich 8093, Switzerland; § Department of Food Science and Technology, Faculty of Science, National University of Singapore, 2 Science Drive 2, Singapore 117542, Singapore; ∥ Technology and Society Laboratory, Empa-Swiss Federal Laboratories for Materials Science and Technology, St. Gallen CH-9014, Switzerland; ⊥ University of Southern Denmark, Department of Green Technology & Danish Institute for Advanced Study, Odense 5230, Denmark

**Keywords:** sustainable chemistry, life cycle assessment, safe and sustainable by design, carbon footprints

## Abstract

The chemical industry is a major contributor to global CO_2_ emissions, accounting for ∼10% of total greenhouse gases, underscoring the need for a sustainability transition. However, public carbon footprint data exists for only hundreds of the more than 100,000 chemicals in commerce, limiting effective action. To address this, we collect high-quality primary chemical carbon footprint data and refine a machine learning model to estimate the carbon footprint of 94,697 chemicals in commerce across 26 national and regional chemical inventories. Our analysis reveals clear molecular structure–carbon footprint relationships that may guide future molecular design: carbon footprints are strongly linked to molecular frameworks, the number of ring structures, and types of heteroatoms. Newly commercialized chemicals tend to have higher carbon footprints than older chemicals, emphasizing the urgent need to embed “sustainable-by-design” principles into chemical innovation from the outset. This study provides a database of chemical carbon footprints and actionable insights for the sustainable design and use of chemicals, supporting the environmental decision-making of chemical producers, consumers, and policymakers.

## Graphical abstract

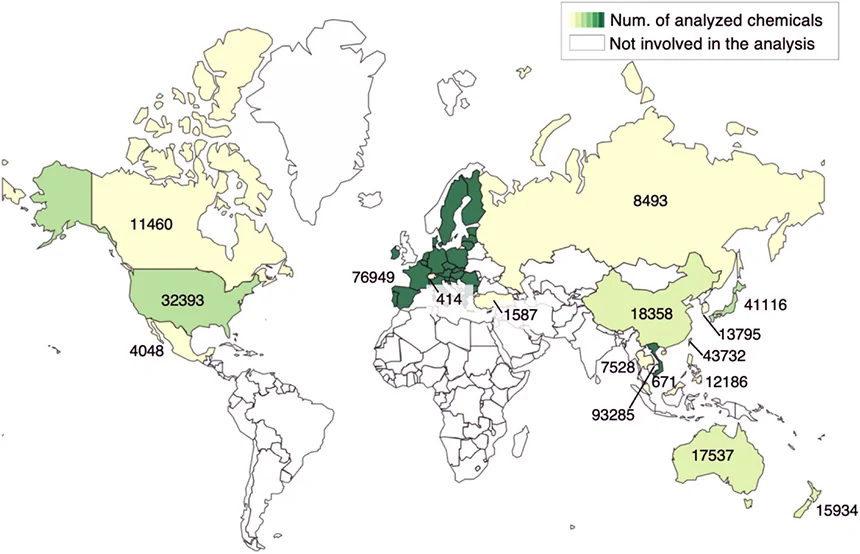

## 1 Introduction

The chemical industry is the third-largest industrial contributor to direct CO_2_ emissions while serving as a cornerstone of modern society, with applications spanning from plastics and agriculture to electronics and pharmaceuticals.^1,2^ As defossilization becomes increasingly urgent, a sustainable transformation of the chemical industry is both necessary and inevitable. Multiple studies have examined potential pathways to achieve net-zero emissions;^2–6^ however, these analyses have largely considered the sector as a whole at the macro level, with a focus on a narrow group of high-production-volume chemicals.

Meanwhile, optimizing defossilization strategies at the company or subsector level remains challenging due to the lack of carbon footprint data for most industry-scale chemicals.^7^ Of over 100,000 registered chemicals, carbon footprint data are publicly available for only a small fraction.^8,9^ In the absence of such data, stakeholders are often forced to rely on proxies or oversimplified estimates that fail to accurately reflect the true carbon emissions associated with chemical production.^9–11^ This deficiency may result in misinformed decisions in product design, regulatory assessments, and transition strategy development, thereby hindering progress toward achieving a sustainable chemical industry and its downstream sectors.

Significant efforts have been made to address these data gaps in the carbon footprint of individual chemicals. Ideally, detailed production process data would be available to enable accurate estimation, but challenges such as confidentiality and limited measurements of reaction inputs hinder this approach.^12–16^ As a result, models and proxy indicators have been used to estimate chemical carbon footprints and bridge data gaps. For instance, simple indicators such as molecular weight and market prices assume that more complex or expensive chemicals have higher carbon footprints.^10,17–19^ More advanced approaches, including machine learning-based tools such as FineChem,^20,21^ APPROPRIATE,^22^ and the COSMO-based SLCA,^23^ have also been developed to improve predictions by integrating additional information such as molecular structures, thermodynamics, and/or simulated production processes. Among these, molecular structure-based models have gained significant attention due to the easy accessibility of molecular structure data, allowing for assessments of a wide variety of chemicals, whereas process-based approaches become more applicable as process information becomes available.^24,25^ In a previous study, we developed FineChem2, a machine learning-based regression model that significantly improves predictive performance over previous models, thanks to advanced algorithms and a more comprehensive training data set.^20^ However, like other models, it was limited to neutral organic chemicals and could not account for their salts, which are the common form of many commercially available chemicals,^26^ such as pharmaceuticals, pesticides, dyes, and specialty chemicals, that would otherwise be too reactive, unstable, or hard to handle. This limitation restricted the applicability of previous molecular structure-based carbon footprint prediction models. Additionally, existing models are either not accessible or not user-friendly, creating barriers to application.

In this study, we introduce a new version of FineChem2 (namely FineChem 2.1), which incorporates enhanced machine-learning approaches, a new algorithm for organic salts and extended training data with harmonized and cleaned data sets of 755 unique chemicals (mainly primary data from the chemical industry)—significantly expanding the model’s applicability domain and improving its predictive power compared to earlier versions. Specifically, it first classifies a given chemical as either having a high carbon footprint (above 10 kg CO_2_-eq/kg) or not, and then applies a regression model to estimate the carbon footprint for those below this threshold. Building upon the updated model, we present FineChemDB—a comprehensive database comprising carbon footprint estimates for over 94,000 industrial-scale organic chemicals, with identifiable structures that fall within the model’s applicability domain to provide readily accessible estimates for chemicals that are already in commerce. We compare the carbon footprint of different generations of chemicals to explore temporal trends in molecular design for sustainability. Furthermore, we identify key molecular features that significantly influence chemical carbon footprints, offering novel insights into sustainable molecular design. Together, FineChemDB and FineChem 2.1 provide researchers, policymakers, and industry with the necessary resources to support a sustainable transition of the chemical industry at the company, country, subsector, and sector levels.

## 2 Methods

### 2.1 Collection of Chemical Data

To establish a comprehensive data set on the carbon footprint of chemicals, high-quality chemical carbon footprint data provided by the industry (over 70%), supplemented with data from ecoinvent v3.8,^27^ IDEA v2.3,^28^ and a previous study^29^ were integrated (Extended content S1, Figure S1). Data cleaning and adjustment were conducted in line with our previously proposed methodologies.^20^ For example, ecoinvent data sets contaminated with direct proxy data, or with heavy reliance on proxies for their precursors, were excluded.^9^ In the case of IDEA and ecoinvent data sets, carbon footprints were refined by incorporating cradle-to-gate data starting from raw material extraction (such as oil or natural gas) of the respective database background system through to the production of the chemical (e.g., ethylene). Energy consumption data was harmonized as described earlier.^20^ Carbon footprint calculations were based on the global warming indicator (100-year) as defined by the IPCC 2021.^30^ Chemical molecular structures were retrieved from PubChem^31^ and stored in the canonical simplified molecular input line entry system (SMILES) format for standardized representation.

To facilitate a comprehensive analysis of chemicals in commerce, chemical registration data from 26 national and regional chemical inventories were retrieved. Detailed data collection methods and sources are outlined in the Supporting Information (Extended content S2 and S3). Unknown or variable composition, complex reaction products or biological materials (UVCBs), mixtures, and polymers were excluded from the analysis due to the absence of clear molecular structures, which renders them incompatible with the assessment model, along with inorganics and organometallics/metalorganics.

### 2.2 Prediction of Chemicals’ Carbon Footprints with the New Pipeline

To make quantitative predictions of the chemical carbon footprints, the initial version of the FineChem2 model^20^ based on a high-quality data set of 755 organic chemicals (over 90% purity) was updated using an enhanced pipeline (FineChem 2.1). Specifically, to improve prediction accuracy for high-carbon-footprint chemicals, a dual-model approach was introduced. This approach begins with a classifier that categorizes chemicals into high-carbon-footprint (above 10 kg CO_2_-eq/kg) and those with carbon footprints of below or equal to 10 kg CO_2_-eq/kg groups based on our previously proposed ML framework.^20^ The threshold of 10 kg CO_2_-eq/kg was selected as a cutoff based on the empirical distribution of carbon footprints of chemicals in our life cycle inventory data set. In the training data set of chemicals with known carbon footprint, the majority (>80%) of industrial organic chemicals cluster below 10 kg CO_2_-eq/kg, while chemicals exceeding this level form a long-tailed distribution characterized by substantially higher and more variable emission intensities, in some cases reaching several hundred or thousands of kg CO_2_-eq/kg.^29^ Regression models tend to perform poorly when attempting to simultaneously fit both the dense low-to-moderate range and the sparse extreme high-end tail. Alternative thresholds (e.g., 15 and 20 kg CO_2_-eq/kg) were also evaluated during model development but resulted in lower predictive performance for both the classifier and the regression model. Therefore, the 10 kg CO_2_-eq/kg threshold was adopted as a pragmatic modeling boundary to improve predictive stability and avoid distortion caused by extreme outliers.

The data set was randomly divided into training, validation, and test sets, with proportions of 80%, 10%, and 10%, respectively. Model performance on the test set was evaluated using micro and macro ROC-AUC scores to assess predictive capability. The confusion matrix was created using the scikit-learn package (https://scikit-learn.org/) to provide a detailed overview of the model’s classification accuracy. For high-carbon-footprint chemicals, the model assigns a high-carbon-footprint label rather than providing an exact prediction. The binary classifier achieved strong performance with an accuracy of 0.93, ROC-AUC of 0.91, and F1 score of 0.87. For chemicals in the test set that have not been seen by the model, the classifier correctly identified over 95% of chemicals with carbon footprints of below or equal to 10 kg CO_2_-eq/kg and 84% of high carbon footprint chemicals (Figure S2a).

A regression model was developed for a subset of chemicals with carbon footprints of below or equal to 10 kg CO_2_-eq/kg, utilizing the same ML framework. The model’s predictive accuracy was evaluated by calculating the root-mean-square error on the test set. For chemicals predicted with carbon footprints of below or equal to 10 kg CO_2_-eq/kg, a regression model was applied to estimate quantitative carbon footprints. On 340 tested cases (68 cases multiplied by five folds), the coefficient of determination (r^2^) and the Spearman correlation coefficient (r) between prediction values and true carbon footprints reached 0.51 and 0.63, respectively (Figure S2b). Approximately half (49%) of quantifiable predictions fell within a 10% error range and 73% within a 30% error range, with a root-mean-square error of 2.36 kg CO_2_-eq/kg. To enhance model performance, hyperparameters were optimized using Bayesian optimization, similar to our prior study.^20^

To enable carbon footprint prediction for organic salts, particularly those with inorganic ions, we developed a hybrid pipeline that combines FineChem 2.1 predictions for organic components with proxy-based estimates for inorganic ions, accounting for reactant variability and associated uncertainty. Detailed methods are provided in the Supporting Information (Extended content S6, Figure S3, and Table S1). This approach can cover over 92% of chemicals collected from the national inventories with ionic constituents (Figure S4).

To further enhance the reliability of predicted carbon footprints, an ensemble-based approach to evaluating uncertainty using the Chemprop package^32^ was implemented. Instead of relying on a single model’s output, predictions were run using five submodels that make independent predictions. Each submodel was built using a different random split of the data set into training, validation, and test sets. The variance among these submodel outputs is then computed and reported alongside the model’s predicted value. This variance serves as an uncertainty metric: a low variance suggests the models agree and the prediction is more reliable, while a high variance indicates greater uncertainty.

### 2.3 Prediction of the Carbon Footprints of Chemicals in Commerce

Before deploying FineChem 2.1 to estimate the carbon footprints of chemicals in commerce, its applicability domain was evaluated using a previously reported approach based on Euclidean distance and k-nearest neighbors.^33,34^ Prior to the applicability domain calculation, hydrogen atoms, metal atoms, and charge were removed, and molecular structures were standardized using RDKit.^35^ Additionally, chemicals were screened for their elemental compositions, ensuring compatibility with elements well-represented in the training data set. Those chemicals with organic parts containing elements other than carbon, oxygen, nitrogen, chlorine, bromine, iodine, fluorine, phosphorus, sulfur, and boron were excluded from the assessment. Additionally, chemicals with inorganic ions not represented in the predefined proxy list (Table S1) were removed from the analysis.

### 2.4 Analysis of the Correlation between Carbon Footprints and Molecular Characteristics

The molecular frameworks and elemental compositions of chemicals were analyzed according to the method defined in RDKit.^35^ To identify molecular frameworks, all nonring systems were removed, except when necessary to connect two-ring structures.^36^ Additionally, heteroatoms within frameworks were replaced with carbon atoms, and double bonds were standardized to single bonds. Following RDKit’s recommendations, the first atom of any exobonded substituent was retained. This abstraction was used solely to group chemicals into shared scaffold classes for interpretation of structure–carbon footprint relationships and was not used as input to the FineChem 2.1 prediction model. Consequently, chemicals sharing the same molecular framework still differed in their functional groups, elemental compositions, and predicted carbon footprints, and the subsequent analyses were based on the distributions within each framework rather than assuming identical properties. Chemicals were grouped according to distinct molecular characteristics (e.g., molecular frameworks, functional groups, and elemental compositions), and for each group, the median carbon footprint values, 95% confidence intervals, and the proportion of high-carbon-footprint chemicals were calculated.

Functional groups were identified using the ExMol toolkit.^37^ To identify key molecular frameworks and functional groups associated with a high carbon footprint, two selection criteria were applied: (1) a likelihood of contributing to a high carbon footprint exceeding 80%, and (2) occurrence in more than 100 chemicals in FineChemDB to ensure representativeness and reduce bias. Unless otherwise stated, all carbon footprint values used in the structural analyses were obtained from an updated FineChem 2.1 model trained on harmonized cradle-to-gate data sets (primarily applying mass allocation; see Extended content S1).

### 2.5 Comparison of the Carbon Footprint of Old-Generation and Newer-Generation Chemicals

Chemicals were categorized based on their registration status in chemical inventories at the time of their initial inclusion. Those already on the market when the inventories were first established were classified as “old-generation chemicals,” reflecting substances that were in commercial use or known to regulatory agencies at the time. In contrast, chemicals added to inventories after the initial release were labeled as “newer-generation chemicals,” representing newer entries that emerged through recent innovation, commercialization, or regulatory submission. For each group, the 95% confidence intervals for chemicals with quantifiable carbon footprints and the proportion of high-carbon-footprint chemicals were calculated.

For most inventories, explicit registration dates for individual chemicals are not publicly available. Therefore, the classification relies on whether a chemical was already listed at the time of inventory establishment versus added in subsequent updates. Consequently, the terms “old-generation” and “newer-generation” should be interpreted as inventory-based categories. This approach serves as a proxy for market presence timing rather than an exact indicator of first commercialization. Differences in regulatory systems across jurisdictions may influence when a chemical appears in a given inventory. As a result, the apparent “marketing” of a chemical in one jurisdiction may not precisely correspond to its global commercialization timeline. To mitigate this limitation, we adopted a rule whereby a chemical classified as “old-generation” in any inventory is treated as old-generation in the global analysis, thereby prioritizing the earliest observable market presence. This reduces the likelihood of misclassifying long-standing chemicals as newer-generation due to administrative or reporting differences across jurisdictions.

Of the chemical inventories analyzed, the Canada and New Zealand ones include registration dates, allowing for analyzing their domestic temporal trends in the carbon footprints of chemicals. In this analysis, newer-generation chemicals in these inventories were categorized into three groups based on their registration dates: before 2011, between 2011 and 2018, and after 2018, and the differences in the carbon footprint of chemicals were compared. This analysis focused solely on newer-generation chemicals, excluding those old-generation ones that were already present in the databases at the time of their release.

### 2.6 Analysis of the Correlation between Carbon Footprints and Other Simple Indicators

The molecular weights of the chemicals were calculated using the RDKit package, and synthetic accessibility scores were determined following a previously reported method.^38^ Synthetic accessibility characterizes chemicals as a score between 1 (easy to synthesize) and 10 (very difficult to synthesize) and is often assumed to reflect the complexity of chemical synthesis that contributes to carbon emissions from chemical production. In this analysis, the reported carbon footprints of chemicals in the training data set were used, except that chemicals with carbon footprints exceeding 10 kg CO_2_-eq/kg were excluded from the analysis to mitigate the influence of outliers and long-tail distribution. Given that the distribution of chemical carbon footprints may deviate from normality, Spearman’s correlation coefficient was used to assess the correlation between carbon footprints and individual indicators.

Price information was sourced from a prior study^39^ as well as the MOLBASE database.^40^ To ensure consistency and comparability, all prices were standardized and converted to Euros. The corresponding carbon footprints of these chemicals were estimated using the FineChem 2.1 model. To reduce the influence of obvious pricing anomalies and noncomparable quotations in the online marketplace, we excluded entries with reported prices above 200 €/kg or below 1 €/kg. These thresholds were selected solely for data cleaning to exclude implausibly high or low prices. Such values may arise because some suppliers use placeholder prices instead of actual transaction or quotation prices to maintain product listings while withholding commercial pricing information. They should not be interpreted as representative price limits for industrial chemicals. For each chemical, the median of these prices was used to represent its typical market value.

### 2.7 Online Application of FineChemDB

FineChemDB was built on the Python web development framework Django 2.2. The site’s front end was designed with Bootstrap, jQuery, and JavaScript. A JSME structure editor was included to enable the drawing and editing of molecules. Notably, FineChem 2.1 requires only the molecular structure—readily provided as a SMILES string or through drawing with the JSME structure editor—as input, making it accessible to a broad range of users. It eliminates the need for coding expertise, which is often unavailable to chemists and industry practitioners. The prediction uncertainty was categorized into three levels and presented in the online tool, namely low (<10% uncertainty), moderate (10–20%), and high (>20%) (Figure S5).

### 2.8 Statistical Information

Two-tailed Welch’s *t* tests were conducted to assess the statistical significance of differences (e.g., on carbon footprint, molecular weights, etc.) between the chemicals in different groups (e.g., with different molecular frameworks, functional groups, or element composition) using the SciPy package (https://scipy.org/). Spearman’s correlation coefficient was used to assess the correlation between chemicals’ carbon footprints and other indicators using the SciPy package (https://scipy.org/), given that the distribution of chemical carbon footprints and physicochemical properties may deviate from normality.

The comparison of the overall differences among molecular characteristics (e.g., molecular frameworks, functional groups, and elemental compositions) was evaluated using the Kruskal–Wallis H-statistic. To make the Kruskal–Wallis H-statistic values standardized and comparable across tasks, epsilon squared (ε^2^) derived from the H-statistic was used for comparison.

$$\varepsilon^{2}=\frac{H-k+1}{n-k}$$

where H is the Kruskal–Wallis statistic, k is the number of groups, and n is the total number of samples.

## 3 Results and Discussion

### 3.1 Overview of the Carbon Footprint of Industrial-Scale Organic Chemicals in FineChemDB

For FineChemDB, 131,765 unique organic chemicals with recognizable structures are initially identified, drawing from 26 national and regional chemical inventories^41^ (for an overview of inventory coverage, see Figure S6 and Table S2). Of these, 109,952 (83%) are neutral chemicals, and 21,813 (17%) are salts. 94,697 (72%) chemicals fall within the applicability domain of the updated FineChem 2.1, as determined by a Euclidean distance-based method and additional screening of the atomic compositions (Figure S7, see Methods). Overall, chemicals with common types of molecular frameworks are largely within the applicability domain, except those chemicals with more complex molecular architectures (e.g., those containing a higher number of ring systems or uncommon atomic compositions such as silicon).

Of these 94,697 chemicals, 66,433 (70%) are classified as likely having a carbon footprint below or equal to 10 kg CO_2_-eq/kg, while the remaining 28,264 (30%) are categorized as high-carbon-footprint chemicals with a carbon footprint above 10 kg CO_2_-eq/kg (Figure 1a, see *Methods*). The threshold of 10 kg CO_2_-eq/kg was adopted as a pragmatic modeling boundary because chemicals above this value exhibit a highly skewed, long-tailed distribution that cannot currently be predicted reliably using regression models. FineChemDB enhances the understanding of chemical carbon footprints by addressing the limitations of existing assessments, which have largely focused on a narrow subset of chemicals with well-characterized life-cycle inventories.

**Figure 1:**
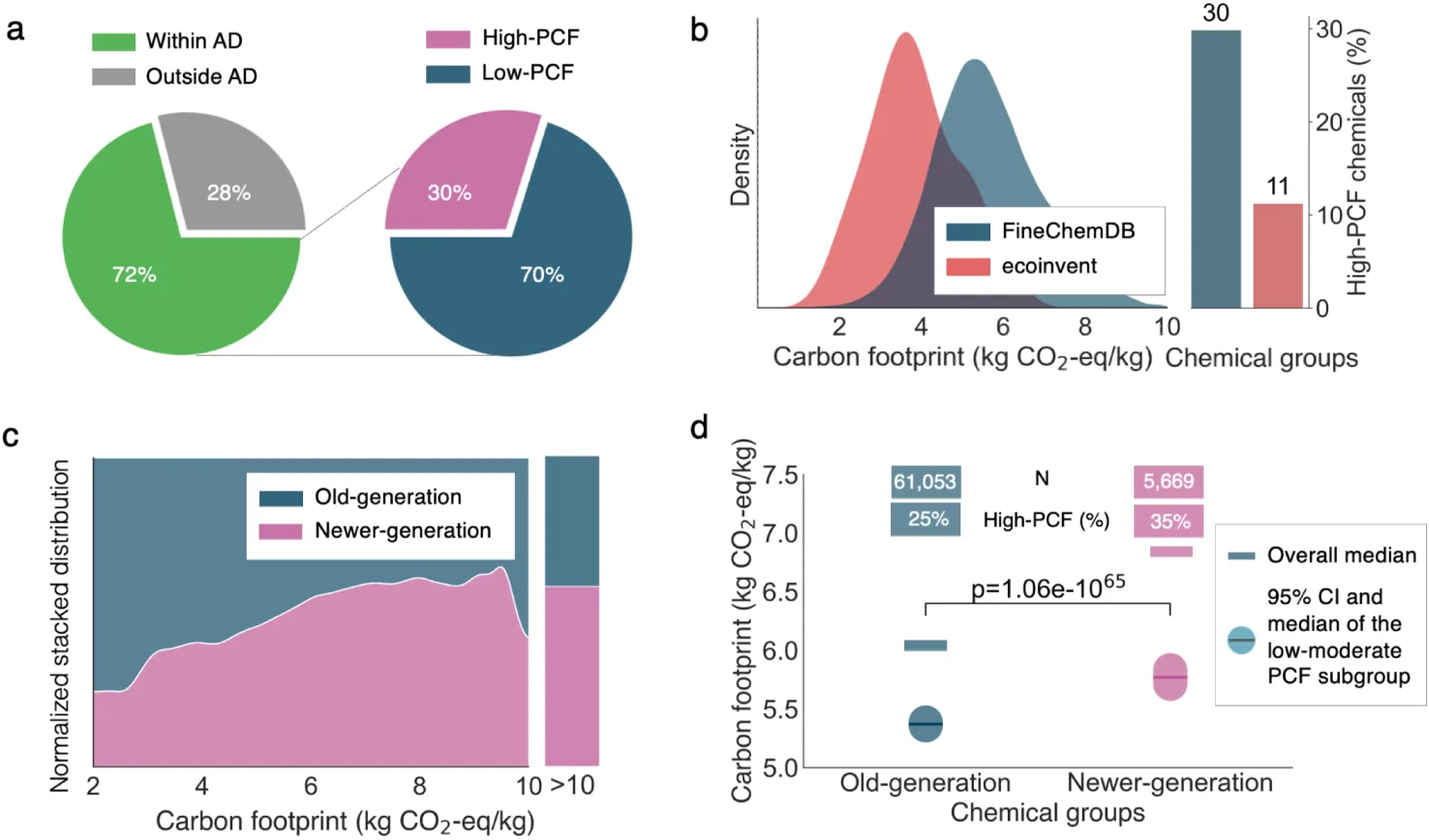
Overview of the carbon footprint of chemicals in FineChemDB. (a) The proportion of chemicals falling within or outside the model’s applicability domain (AD), and the proportion with high vs low-moderate product carbon footprint (PCF). (b) Comparison of the probability density distributions of product carbon footprints between FineChemDB and the subset of approximately 170 pure organic chemical with clear structure production data sets retrieved from the ecoinvent v3.8^28^ life cycle inventory database. Probability densities were estimated using kernel density estimation and normalized so that the area under each curve equals one. (c) Normalized stacked distribution of carbon footprint values for the old-generation and newer-generation chemicals. High-PCF chemicals are shown as one column under *>10*. (d) Comparison of the carbon footprint of old-generation and newer-generation chemicals. *High PCF (%)* denotes the fraction of chemicals with carbon footprints above 10 kg CO_2_-eq/kg, excluding those that fall outside the model’s AD. *N* denotes the number of chemicals. *P* values were calculated using two-tailed Welch’s *t* tests.

Compared with existing life cycle inventory databases, such as ecoinvent v3.8 ^28^, which contains carbon footprint data for approximately 170 pure organic chemicals with clear molecular structure, FineChemDB substantially expands the coverage of chemicals with carbon footprint estimates and represents a much broader range of chemicals currently in commerce (see Figure 1b for a comparison of the distributions). High-carbon-footprint chemicals have been registered in all jurisdictions investigated, though the proportions vary (Figure S8). For an overview of the updated FineChem 2.1’s enhanced predictive performance relative to earlier methodologies, see Tables S3 and S4). Specifically, FineChem 2.1 achieved the highest regression performance among all evaluated molecular structure-based models (r^2^ = 0.51 ± 0.13), substantially outperforming conventional fingerprint-based baselines, while also achieving the highest classification accuracy (0.93 ± 0.05).

### 3.2 Newer-Generation Chemicals Exhibit a Higher Carbon Footprint than Older-Generation Ones

Using the registration status of individual chemicals in the inventories,^41^ we distinguish chemicals present on the market before the inventories were established as “old-generation chemicals” and those added after the creation of the inventories as “newer-generation chemicals” (See *Methods*). This allows us to compare their carbon footprints and, thus, get insights into potential temporal trends in molecular design for sustainability.

Newer-generation chemicals have a higher carbon footprint than old-generation chemicals, indicating a shift in molecular design (Figure 1c and d). This indicates that the chemical industry has increasingly developed high-performance chemicals with more complex molecular structures or more heteroatoms (Figure S9) for specialized functions and higher market value,^42,43^ but at the cost of higher carbon footprints. Their complex structures and higher heteroatom content often require harsher reaction conditions, specialized reagents, and additional synthesis steps, resulting in increased energy and material use, thereby elevating their associated carbon footprints.^44^ In addition, unlike older chemicals produced in optimized, large-scale, and integrated systems, newer ones are often made at smaller scales with less mature manufacturing procedures and weaker process integration, limiting efficiency. These trade-offs should be noted and carefully evaluated in the development of sustainable chemicals from the initial stages of design.

Of the reviewed chemical inventories, only Canada and New Zealand include registration dates, enabling analysis of their domestic temporal trends. While the New Zealand inventory shows a rising trend in carbon footprints over time, the Canadian inventory does not (Figure S10). This divergence suggests factors such as regulatory approaches, industrial landscape and priorities, and/or market dynamics may drive different trends in the carbon footprint of newly introduced chemicals. This warrants more in-depth analysis in future studies.

To further illustrate the implications of FineChemDB, we conducted an additional case study on the 2016–2019 U.S. Chemical Data Reporting data set. This analysis shows that, although high-carbon-footprint chemicals generally account for a small fraction of total chemical input volumes across most industrial sectors, their contribution to the overall embedded emissions can be disproportionately large in some sectors, highlighting the potential hotspots and the value of targeted substitution strategies. More details on the case study are provided in Extended content S5 and Tables S5 and S6.

### 3.3 Molecular Frameworks Largely Influence the Carbon Footprint of Chemicals

Using FineChemDB, we have further investigated the relationship between molecular structures and carbon footprints, aiming to provide further insights for sustainable-by-design. Specifically, we have examined four key characteristics: the molecular frameworks (the core structural backbone of a molecule, excluding side chains and replacing double bonds with single bonds and heteroatoms with carbon atoms), number of ring structures, functional groups, and elemental compositions (See *Methods*).

From the over 94,600 chemicals that are within the applicability domain of FineChem 2.1, we identify 3,759 distinct types of molecular frameworks, highlighting the significant structural diversity in the global market. Notably, 29% of these chemicals are acyclic, lacking ring structures. The six most common molecular frameworks are cyclohexane, cyclopentane, dicyclohexylmethane, decahydronaphthalene, 1,1′-bi­(cyclohexane), and octahydro-1*H*-indene, accounting for 69% of the chemicals analyzed (Figure 2a).

**Figure 2:**
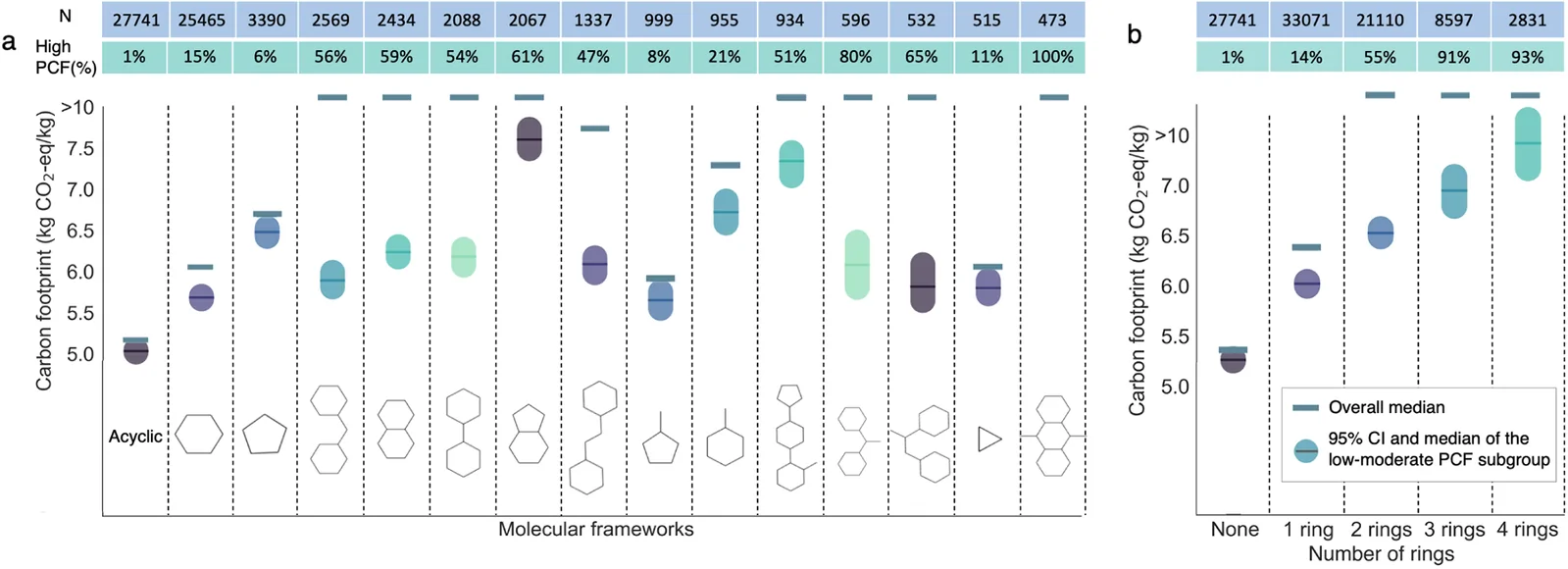
Carbon footprints of chemicals (a) with varied molecular frameworks and (b) with different numbers of rings. The lines indicate the median carbon footprints of all chemicals within each group, including both low-moderate and high carbon footprint compounds. *N* denotes the number of chemicals within the respective groups, and *High PCF (%)* is the fraction of chemicals with carbon footprints above 10 kg CO_2_-eq/kg.

Our analysis reveals significant variations in carbon footprints across chemicals with different molecular frameworks (Figure 2a). In particular, the carbon footprint clearly increases with the number of rings in the molecular structure (Figure 2b). Acyclic chemicals generally have lower carbon footprints, with only 1% showing carbon footprints above 10 kg CO_2_-eq/kg. This suggests that incorporating ring structures may contribute to a higher carbon footprint, even for simple ring structures such as cyclohexane and cyclopentane. One possible explanation, supported by previous chemical engineering and LCA studies, is that ring-forming reactions often require multiple synthetic steps, harsher reaction conditions, or protecting-group strategies, thereby increasing energy and material requirements; all of which increase energy use and material inputs.^45,46^ Additionally, many industrial ring-containing chemicals are derived from aromatic platform chemicals, whose production may already carry higher embedded emissions than simple acyclic building blocks.^47^ Furthermore, ring structures containing heteroatoms frequently need to be built up in energy-demanding processes such as condensations and may not allow the use of simple ring structures (e.g., benzene) that are available at lower carbon footprints, for example, from the catalytic reforming or steam cracking of fossil feedstocks.

Acyclic chemicals, in contrast, can often be synthesized from more simple acyclic building blocks that are available at relatively low carbon footprints. Even for more complex, longer-chain acyclic compounds, hydroformylation or oligomerization reactions (e.g., Ziegler-Alfol synthesis) may enable their formation with relatively low carbon footprints.^10^ Another possible explanation, is that certain acyclic compounds can also be synthesized from biogenic feedstocks such as vegetable oils through processes including saponification or transesterification of triglycerides, which may be associated with a comparatively lower carbon footprint under some production systems. However, biogenic feedstocks can trigger other environmental impacts, such as land-related biodiversity loss or water stress, depending on the type and source of biomass.^48^ These impacts have not been considered in the present paper.

Chemicals with more than two rings, such as dicyclohexylmethane, decahydronaphthalene, or 1,1′-bi­(cyclohexane) moieties, show a higher proportion of having a high carbon footprint, compared to acyclic or monocyclic chemicals. Notably, 80% of the chemicals containing an ethane-1,1-diyldicyclohexane moiety, and 100% of the chemicals with a 9,10-dimethyltetradecahydroanthracene moiety, fall into the high-carbon-footprint category. Basic building blocks containing such ring structures are more commonly available from the distillation of coal tar, which is a byproduct of coke manufacturing. Due to the large number of complex aromatic substances with similar boiling points in the coal tar, as well as the formation of azeotropes, their isolation for further manufacturing of downstream chemical products can be energy-demanding, which then results in elevated carbon footprints. Should the desired downstream compound also contain heteroatoms, these building blocks may only be partially used, necessitating even more advanced synthesis strategies than those required for simple aromatic structures. As examples of this, we identify 12 other molecular frameworks, each with over an 80% likelihood of contributing to a high carbon footprint (Table S7). It is worth noting that the type of ring structure also plays a role: chemicals with four- or five-membered rings generally have higher carbon footprints than those with three- or six-membered rings (Figure S11). Moreover, those chemicals containing only heterocycles and nonaromatic rings tend to have higher carbon footprints than those with all-carbon or aromatic rings (Figure S11).

Element compositions and functional groups also influence the chemical carbon footprints. Specifically, halogen-containing chemicals tend to exhibit significantly higher carbon footprints, while those composed solely of carbon and hydrogen show a markedly lower carbon footprint (Figure S12). This statistical association may partly reflect the upstream production burden of halogens: for instance, bromine and iodine are partly produced from brines, where the separation and purification of these two compounds is energy demanding, resulting in comparably high carbon footprints. In contrast, chlorine and fluorine (the latter in the form of hydrogen fluoride) are available as raw material for halogenation in chemical syntheses at lower carbon footprints, but can lead to the release of powerful greenhouse gases such as chlorinated hydrocarbons or hydrofluorocarbons. Even if these are released in small quantities during the manufacturing processes, they can contribute to very high carbon footprints per amount of the main product. Moreover, halogenation reactions in industrial synthesis often involve corrosive reagents, high energy demand, and specialized waste treatment steps, all contributing additional greenhouse gas emissions.^49^ Furthermore, among the top 15 most common functional groups, chemicals containing aromatic rings, conjugated double bonds, as well as heterocyclic structures, tend to exhibit higher carbon footprints (Figure S13). Notably, chemicals featuring secondary aromatic amines, diaryl thioethers, or phosphonium groups show more than 80% likelihood of being associated with a high carbon footprint (Table S8). These groups frequently require multistep synthesis, specialized reagents, and/or generate byproducts that reduce atom economy, thereby further increasing the overall energy intensity of production.^50^

Notable differences exist, for example, between different types of heteroatoms. One possible explanation, supported by previous LCA studies, is that the upstream production of these elements differs substantially. Sulfur is often recovered as a low-value byproduct of oil refining, natural gas processing, or metal smelting and therefore may carry a relatively limited burden depending on the allocation approach adopted. In contrast, phosphorus is typically produced through energy-intensive smelting processes, while nitrogen for chemical synthesis is commonly supplied via ammonia production. The carbon footprint of ammonia itself depends strongly on the production route (e.g., conventional natural gas reforming versus emerging low-carbon pathways) and the extent of emission abatement. These upstream differences may partly contribute to the statistical associations observed here, with nitrogen-containing compounds tending to exhibit higher estimated carbon footprints than sulfur- or phosphorus-containing compounds (Figure S12). However, these associations should not be interpreted as universal rules, as they may vary with production technologies, feedstocks, and emission control measures.

Overall, variations based on element compositions and functional groups are less pronounced than those observed for the molecular frameworks and number of ring structures (Figure S14). These observations provide simplified guidance for estimating the carbon footprint of chemicals and for identifying low-carbon-footprint alternatives, starting with the selection of molecular frameworks, followed by fine-tuning functional groups.

### 3.4 Conventional Proxy Indicators Fail to Fully Reflect Chemical Carbon Footprints

In the absence of reliable carbon footprint data, surrogate indicators, such as molecular weight, synthetic accessibility scores that reflect the estimated difficulty of chemical synthesis,^38^ and market price-based estimations, have often been used to estimate carbon footprints. Our analysis reveals a significant but weak correlation between molecular weights and carbon footprints (r = 0.19, p = 5.92e^–7^, Figure 3a). Notably, this correlation was mainly limited to chemicals with relatively low molecular weights (Figure S15a–c). For more complex chemicals, the correlation dissipates, indicating that molecular weight alone is insufficient as a predictor for these cases.

**Figure 3:**
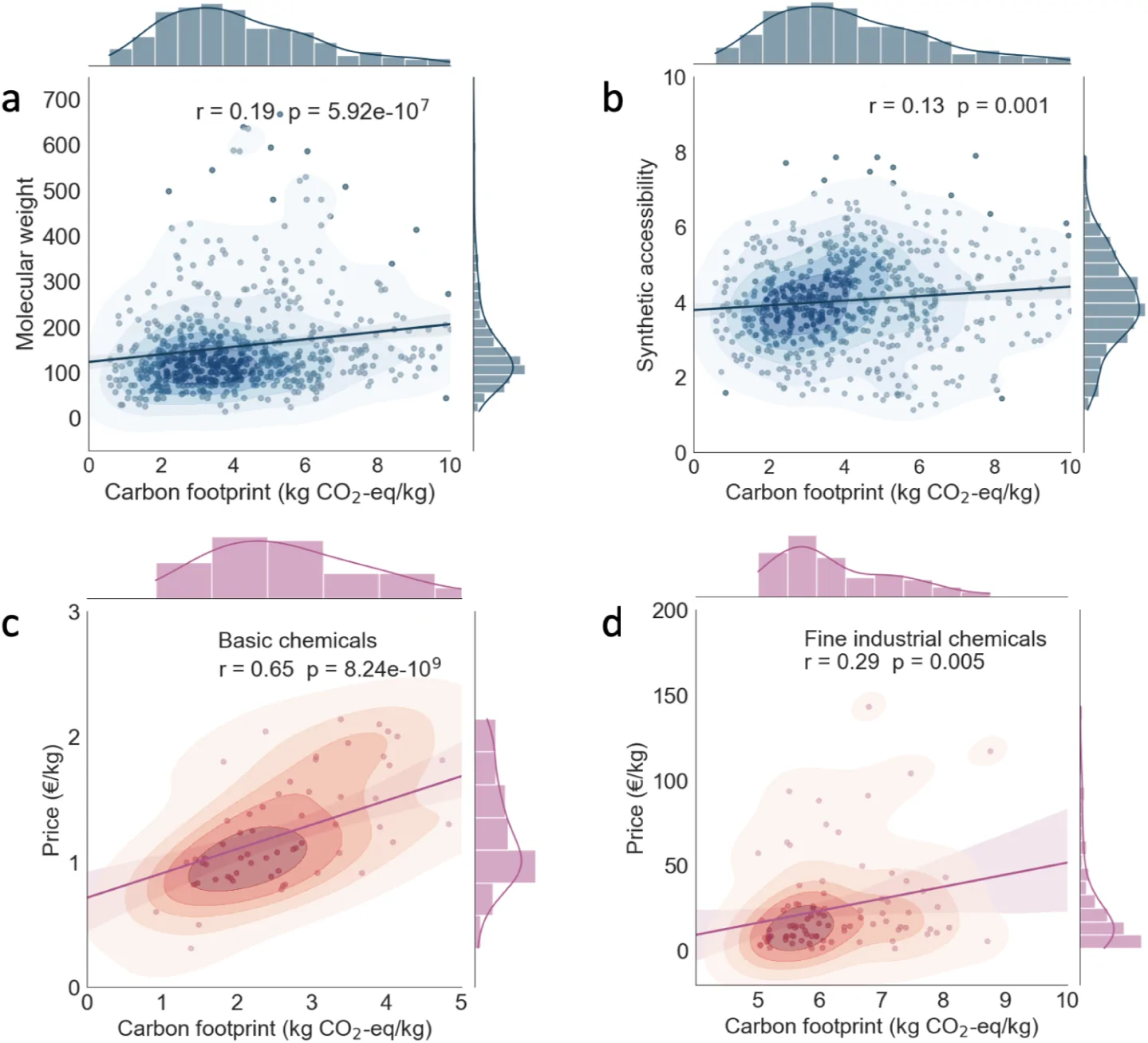
Correlations between chemicals’ carbon footprints and (a) molecular weight (*N* = 618, r = 0.19, *p* = 5.92e^–7^), (b) synthetic accessibility scores (*N* = 618, r = 0.13, *p* = 0.001), (c) market prices of basic chemicals (*N* = 63, r = 0.65, *p* = 8.24e^–9^), and (d) market prices of specialized fine industrial chemicals (*N* = 89, r = 0.29, *p* = 0.005). The r value presents the Spearman correlation coefficient. The spheres show density contours—darker areas mark higher data concentration, lighter ones indicate lower density.

Synthetic accessibility may partially reflect the complexity of chemical synthesis that contributes to carbon emissions in chemical production.^38^ However, our findings indicate that the correlation between chemicals’ synthetic accessibility and carbon footprint is even lower than that of molecular weight (r = 0.13, p = 0.001, Figure 3b). Similar to molecular weight, the high complexity group showed a lower statistically significant correlation (Figure S15d–f).

To explore the accuracy of market price as an indicator, we have examined the relationship between prices and carbon footprints for 63 basic organic chemicals collected from prior studies,^39^ such as acetic acid, acetone, and 1-butanol. A statistically significant and relatively stronger correlation is observed compared to the previous two indicators (r = 0.65, p = 8.24e^–9^, Figure 3c). However, when analyzing 89 randomly collected specialized fine industrial chemicals with carbon footprints between 5 and 10 kg CO_2_-eq/kg collected from the MOLBASE database^40^—including common agricultural chemicals, drug intermediates, and food additives—the correlation weakened significantly (r = 0.29, p = 0.005, Figure 3d).

Collectively, these findings suggest that conventional proxy indicators, such as molecular weight, synthetic accessibility, and market price, have limited predictive value when used individually for broad carbon footprint estimation. Although these indicators may capture certain aspects of production complexity and could contribute to more sophisticated predictive models, our analyses indicate that they are insufficient as standalone proxies across the diverse chemical space investigated in this study. The FineChemDB and FineChem 2.1 prediction model offers a more accurate and scalable solution for estimating the carbon footprint of chemicals than these traditionally applied proxy indicators, supporting more informed decision-making in sustainable chemical design and lifecycle assessment. Notably, FineChemDB requires only the molecular structure as input for search, making it accessible to a broad range of users and eliminating the need for coding expertise, which is often unavailable to chemists and industry practitioners.

### 3.5 Discussion of Alternative Approaches

FineChem 2.1 can be situated within a growing range of chemical LCA data-gap-filling methods, including the taxonomy-based probabilistic proxy approach of Ellingboe et al.,^51^ the COSMO-based structure–impact model of Calvo-Serrano et al.,^23^ the reaction-informed APPROPRIATE framework,^22^ the automated route-resolved CRYSTAL framework,^52^ and the stoichiometry-based and decision-tree methods developed by Langhorst et al.^12^ These methods address different information states, from chemical identity or molecular structure alone to a known reaction equation or production route, and cannot therefore be compared fairly through a single equivalent numerical benchmark. Ellingboe et al.^51^ assign chemicals to ClassyFire/ChemOnt taxonomic groups and use the corresponding empirical impact distributions from the Federal LCA Commons or ecoinvent as probabilistic proxies. This transparently represents within-group variability but does not differentiate between chemicals within the same group, and its results depend strongly on the coverage and representativeness of often sparsely populated classes. FineChem 2.1 instead uses a substantially larger industrial product-carbon-footprint data set to generate chemical-specific estimates with prediction uncertainty and applicability-domain information, although it does not retain the direct interpretability of selecting an observed taxonomic proxy distribution. The COSMO-based approach^23^ predicts nine impact indicators for a data set of 90 chemicals using combinations of molecular descriptors, thermodynamic properties and molecular surface-charge-density distributions. Its physically informed σ-profiles can improve predictions without experimental property data, but the model remains component-specific, was developed from a comparatively small data set and cannot distinguish alternative production routes.^23^ FineChem 2.1 has a narrower focus on product carbon footprints but is calibrated and independently evaluated using a broader industrial data set that includes more complex and higher-impact chemicals. APPROPRIATE moves from component- to process-specific prediction by combining a latent molecular representation with descriptors derived from a known gross reaction equation and Gaussian-process regression, thereby allowing alternative reactions and background scenarios to be distinguished.^22^ However, APPROPRIATE requires the reaction route as input and was trained on only 304 unique reactions for 166 chemicals. Its simplified flowsheeting estimates minimum energy requirements that were found to underestimate actual process energy and contribute relatively little predictive information. CRYSTAL is more process-explicit, predicting retrosynthetic pathways and generating route-resolved inventories by balancing reactions and estimating yields, auxiliary-material use, utilities, emissions and waste flows.^52^ Its estimates of yields, energy demands and auxiliary inputs incorporate the decision-tree models of Langhorst et al., which were trained on a finite data set dominated overlapping with the data used for APPROPRIATE on established, often very large-scale industrial processes. Their transferability to smaller-scale fine and specialty chemical production may consequently be limited. CRYSTAL’s accuracy also depends on whether its automatically generated, largely patent-derived routes, reagents and solvents represent industrial practice, while deviations in route selection, yields, upstream inputs and utilities can accumulate across multistep pathways and may already become substantial after only a few synthesis steps. More generally, the benchmarking by Langhorst et al.^12^ shows that reaction stoichiometry and simplified energy estimates alone cannot adequately capture industrial utility demand, separation, purification and heat integration. FineChem 2.1 is therefore intended primarily for early stage screening, data-scarce applications and gap-filling, whereas route-specific frameworks provide more detailed and interpretable results under the condition that high-quality representative reaction, energy and process information is available.

### 3.6 Critical Appraisal and Outlook

This study presents FineChemDB, a comprehensive database with unprecedented data coverage of over 94,000 organic chemicals in commerce compiled from 26 national and regional inventories. This database is created with our updated machine-learning tool FineChem 2.1, thanks to extensive data collection (including primary industry data), cleaning, and a dual-model framework that integrates classification and regression with robust uncertainty measures. Using in-depth analysis of this comprehensive data set, we have found that about one-third of these chemicals have high carbon footprints, with molecular structures—especially ring systems—playing a key role. Additionally, newer-generation chemicals tend to have higher carbon footprints than older ones, suggesting a trade-off between performance and sustainability that should be carefully considered from the earliest stages of chemical design. Building upon FineChemDB, we provide novel insights in designing low-carbon-footprint chemicals by analyzing molecular frameworks, ring structures, functional groups, and element composition. By illuminating opportunities for sustainable molecular design and production, it lays a foundation for actionable strategies to drive the global chemical industry’s transition toward sustainability. Overall, FineChemDB and FineChem 2.1 fill critical data gaps in screening and assessing the sustainability of chemicals, providing valuable insights and guidance for chemists, chemical producers, downstream industrial users, and policymakers.

At the same time, among the 109,952 organic chemicals in commerce investigated, over 20,000 fall outside the applicability domain of FineChem 2.1, and another 28,264 chemicals are classified as high-carbon-footprint chemicals but are not assigned quantitative carbon footprint values because the currently available data for this class are sparse. Therefore, while FineChemDB already provides unique data coverage, further expansion of the FineChem 2.1 model is needed to expand the coverage of FineChemDB.

The expansion of FineChem 2.1 should begin with enlarging the training data set to improve model accuracy and coverage. Additionally, although the updated model and analysis pipeline have, for the first time, enabled the prediction of over 90% of organic salts collected from chemical inventories, its applicability remains limited to chemicals with common inorganic compositions (e.g., Na^+^, K^+^, NH_4_^+^, Cl^–^, SO_4_^2–^, and PO_4_^3–^; Table S1). Organic salts with rare elements—such as tungsten and rhenium—are not considered due to the lack of reliable life cycle inventory data. Moreover, the introduction of inorganic ions to a chemical structure may result in additional energy requirements during synthesis, purification, or crystallization. However, due to the limited availability of detailed, reaction-specific energy and emission data, such impacts are not explicitly captured in the current modeling framework.

Another key limitation is the current inability to account for chemical purity in the estimates. This would not be an issue of using FineChemDB as a general screening tool, but may have important implications when conducting detailed assessments for high-value applications such as final pharmaceutical and pesticide products. For example, chiral purity, a critical factor for biological activity, toxicity, and regulatory approval, is not explicitly considered by the model. Similarly, pharmaceutical-grade chemicals often require additional processing steps to achieve high purity, including enantiomeric separation, advanced filtration, or recrystallization, all of which may contribute significantly to their carbon footprints. These additional details in production and quality requirements highlight the need for developing more application-specific approaches that can integrate such considerations into FineChemDB (e.g., by introducing additional uncertainty factors) and/or FineChem 2.1 (e.g., by expanding the model’s scope and capabilities).

Additionally, the same chemical produced via different synthesis routes or using different types of feedstocks can exhibit significantly different carbon footprints. For example, ethylene produced from fossil-based naphtha cracking typically has a lower carbon footprint than ethylene derived from coal, while glycerol synthesized from petrochemical routes tends to have a higher carbon footprint compared to biobased routes where it is a byproduct of biodiesel manufacturing. These variations are currently not captured by the model or any other existing tools based on chemical structures alone, and therefore are also not represented in FineChemDB. This limitation is inherent to molecular structure-based estimation because the required production information is not encoded in the molecular structure. To ensure that FineChem 2.1 provides carbon footprint values representing averages across production routes rather than extremes, we calculated a weighted mean carbon footprint of the available production routes in the training data when multiple cradle-to-gate inventories were available for the same chemical. In spite of the lack to account for production-route variability, molecular-structure based models provide actionable tools for incorporating carbon footprint considerations into the early stages of chemical design, procurement, and portfolio screening. They may also contribute one environmental input to broader frameworks such as the EU Safe-and-Sustainable-by-Design (SSbD) initiative, where carbon footprint represents one of multiple criteria alongside safety, toxicity, functionality, circularity, and economic considerations. For instance, product developers can use these tools to screen alternative ingredients and choose those with lower carbon footprints during early stage research and development. Similarly, it may be useful for process optimization, targeting high-carbon-footprint educts and reaction media. In supply chain management, FineChemDB can support preliminary screening, hotspot identification, and prioritization of chemicals for more detailed assessment, particularly where process-specific life cycle inventory data are unavailable. The uncertainty estimates reported by FineChem 2.1 should also be considered when interpreting prediction results. Chemicals with low prediction uncertainty may be suitable for preliminary screening and prioritization, whereas those associated with higher uncertainty should be treated with greater caution and, where decisions have significant environmental or regulatory implications, followed by more detailed process-specific life cycle assessment. To enhance accuracy and relevance for applications requiring higher-resolution data, future model and database development should incorporate more detailed chemical information, such as their synthesis routes and feedstocks. In addition, bulk commodity chemicals such as ethylene, propylene, and methanol are typically covered with more detailed models in existing LCA databases (e.g., ecoinvent, IDEA). Although these data sets may still exhibit substantial variability, they provide a stronger basis for assessment. As such, they should not be the primary targets of predictive analysis, which is instead designed to fill data gaps for the many tens of thousands of other chemicals lacking process-level inventories. Accordingly, FineChemDB is intended to complement rather than replace detailed product life cycle assessment. It is most appropriate for early stage molecular screening, substitution assessment, procurement support, and portfolio-level hotspot identification, whereas product-level environmental assessment and decision-making still require route-specific, site-specific, and supply chain-specific life cycle inventory data.

Beyond tool deployment, our structure–footprint analysis uncovered generalizable patterns that can guide sustainable chemistry. For example, chemicals with certain frameworks and functional groups, with extensive ring systems and halogen atoms, tended to exhibit significantly high carbon footprints. In contrast, small, acyclic molecules with carbon-rich functionalities often corresponded to lower estimated footprints. Such insights can serve as rules of thumb for chemists seeking low-impact design strategies.

We are confident that FineChemDB, together with FineChem 2.1, can improve the granularity and accuracy of environmental assessments and empower stakeholders across the value chain to make data-driven decisions that advance defossilization goals—not just for the chemical industry but the myriad sectors that depend on its outputs. FineChem tool can be accessed via https://esd.ifu.ethz.ch/downloads/finechem2.html.

## Supplementary Material



## Data Availability

Data generated in this research are available in the Supporting Information and in a Zenodo repository (https://zenodo.org/records/15574876). The life-cycle inventory data for chemical production were collected from IDEA v2.3 (https://idea-lca.com/en/), Ecoinvent v3.8 (https://ecoinvent.org/), a previous study (10.1021/acssuschemeng.8b05473), and industrial partners. User licenses are required to access the IDEA and Ecoinvent databases. Primary raw chemical production process data from industrial partners are confidential under a nondisclosure agreement, but all modeled results are openly available. Chemical price data used for analysis were collected from a previous study (10.1002/bbb.1786) and MOLBASE (https://www.molbase.cn/). Data on chemical consumption volume in each industrial sector were collected from the 2016–2019 U.S. Environmental Protection Agency’s Chemical Data Reporting data set (https://www.epa.gov/chemical-data-reporting). Data on chemicals in commerce were collected from 26 inventories listed in the Supporting Information.

## Notes

Detailed methods; supplementary model evaluation and validation; applicability domain analysis; organic salt prediction workflow; uncertainty analysis; supplementary analyses of molecular structural features and chemical inventories; additional descriptions (S1–S6); additional figures (Figures S1–S15); additional tables (Tables S1–S8); supplementary references (PDF)
