# Supplementary material for: Mapping the Carbon Footprint of Organic Chemicals in Commerce
Source: Environ Sci Technol. 2026 Oct 28;60(44):31710–20. doi: 10.1021/acs.est.6c06036 (PMC13757936; doi:10.1021/acs.est.6c06036)
Supplement: Supplementary file 1 [file es6c06036_si_001.pdf]

Supporting information

Mapping the Carbon Footprint of Organic Chemicals in Commerce

Dachuan Zhang[1,2,3*], Zhanyun Wang[2,4,5], Christopher Oberschelp[1, 2], Jing Huo[1, 2], and Stefanie Hellweg[1, 2,*]

[1] Ecological Systems Design, Institute of Environmental Engineering, ETH Zürich, Zürich 8093, Switzerland

[2] National Centres of Competence in Research (NCCR) Catalysis, Switzerland

[3] Department of Food Science and Technology, Faculty of Science, National University of Singapore, 117542, Singapore

[4] Technology and Society Laboratory, Empa-Swiss Federal Laboratories for Materials Science and Technology, St. Gallen CH-9014, Switzerland

[5] University of Southern Denmark, Department of Green Technology & Danish Institute for Advanced Study, 5230 Odense, Denmark

[*] To whom correspondence should be addressed:

Dachuan Zhang, dachuan.zhang@nus.edu.sg

Stefanie Hellweg, stefanie.hellweg@ifu.baug.ethz.ch

This file includes:

Number of pages S1-S33

Extended content S1-S6

Extended figures S1-S15

Extended tables S1-S8

Extended content S1. Collection of chemical carbon footprint data from industry

The industry-provided data were mainly representative of European production conditions and were based on primary process information collected from chemical producers. For confidentiality reasons, individual process inventories, production sites, and company-specific datasets cannot be disclosed. All datasets were checked before inclusion. We retained only datasets based on primary production information and excluded records that relied on proxy data for the target chemical or on key upstream supply-chain processes dominated by proxy inventories. Additional checks included consistency of chemical identity based on SMILES and the original documents, and system boundary. For multi-output processes, the native allocation approach was followed, which was in the overwhelming majority of cases mass allocation. The data covers manufacturing plants from all major manufacturing regions globally and with a wide variety of manufacturing pathways, but certain manufacturing locations (such as China) or manufacturing pathways (such as from coal) may be underrepresented in comparison to their global market share. The data also contains a small but relevant share of biogenic feedstock-derived manufacturing routes.

Extended content S2. Collection of chemicals in commerce from national chemical inventories

The list of national chemical inventories was compiled based on the database developed by Wang et al., with additional updates incorporating newly released and recently revised inventories (**Table S2**). Some inventories, such as the Mexican National Inventory of Chemical Substances, were obtained through private correspondence.

Since most inventories report chemical identities using CAS Registry Numbers (CASRN), we adopted CASRN as the standard identifier to track unique chemicals across different sources. As a first step in processing these inventories, we used the STDNUM package in Python (https://pypi.org/project/python-stdnum/) to validate the format of reported CASRNs and identify any inconsistencies. For chemicals reported with an EC number instead of a valid CASRN, we searched the European Chemicals Agency database (https://echa.europa.eu/information-on-chemicals) to find the corresponding CASRN. If an EC number did not match any CASRN in the European Chemicals Agency database, that entry was excluded from further analysis. Some records lacked CAS numbers and were reported only with chemical names. However, processing chemical names on a large scale is challenging due to minor formatting inconsistencies (e.g., extra spaces, italicization, or regional spelling differences such as "z" vs "s"). These inconsistencies can prevent accurate mapping of a chemical name to its assigned CASRN, so such entries were also excluded from the analysis. Additionally, some records were identified only by internal

proprietary identifiers or masked for confidentiality reasons, making it impossible to retrieve their CASRNs. As a result, these entries were excluded from the analysis.

To enhance identifier coverage, we queried multiple public databases to retrieve simplified molecular input line entry system (SMILES) representations for these chemicals. The primary sources included PubChem (https://pubchem.ncbi.nlm.nih.gov/), the EPA CompTox Dashboard (https://comptox.epa.gov/dashboard/), the OECD QSAR Toolbox (https://qsartoolbox.org/), and SMILES_CAS, a dataset linking CASRN to SMILES from the OPERA QSAR platform. Given that these databases vary in curation quality, a SMILES string was only accepted if it matched across all four sources or if it matched between at least one public database and the Elsevier Reaxys database (https://www.elsevier.com/products/reaxys). For compounds without matched SMILES in these sources, we manually retrieved SMILES data from SciFinder (https://www.cas.org/solutions/cas-scifinder-discovery-platform/cas-scifinder).

Extended content S3. Global landscape of organic chemicals in commerce

Figure S6 presents a global map illustrating the number of registered chemicals across different countries and regions. The numbers on the map represent the count of registered chemicals in each respective country. The map employs a color gradient (shades of green) to indicate the number of registered chemicals, with darker green representing a higher number of registered chemicals, while white-colored regions indicate countries not included in the analysis. Vietnam (93,285 chemicals) has the highest number of registered chemicals among the countries analyzed. The European Union (76,949 chemicals) also has a large number of registered chemicals, reflecting its comprehensive chemical regulatory framework. China (18,358 chemicals), Japan (41,116 chemicals), Taiwan (43,732 chemicals), and the United States (32,393 chemicals) also show significant numbers of registered chemicals. Canada (11,460 chemicals), Russia (8,493 chemicals), and Thailand (7,528 chemicals) fall within the moderate range. Some countries, such as Turkey (1,587 chemicals), Switzerland (414 chemicals), and Malaysia (671 chemicals), report a lower number of registered chemicals. Countries that are uncolored (white) were not included in the dataset, meaning their chemical inventories were either unavailable, inaccessible, or not considered in this study.

Extended content S4. Comparison of FineChem 2 with baseline models and tools

To benchmark the performance of the proposed model, a set of baseline machine learning (ML) models was constructed by combining three widely used algorithms—artificial neural networks (ANN), random forests (RF), and support vector machines (SVM)—with three types of molecular descriptors, including MACCS keys, RDKit fingerprints, and extended-connectivity fingerprints (ECFP4. Model evaluation was

conducted using two complementary data splitting strategies to assess both interpolation and extrapolation performance. In the random splitting strategy, the dataset was randomly divided into training and test sets at a ratio of 9:1, and hyperparameter optimization was performed using five-fold cross-validation on the training set in conjunction with grid search. For RF models, the number of estimators (10–1000, step size 200), minimum samples required for node splitting (1–100, step size 20), and minimum samples per leaf (2–10, step size 2) were optimized. For ANN models, hidden layer configurations ([100] and (100, 30)) and solver types (Adam, SGD, and LBFGS) were explored. For SVM models, kernel type (linear and RBF), regularization parameter (C = 1, 10, 100, 1000), and gamma (10^{-3} and 10^{-4}) were tuned. All ML models were implemented using scikit-learn (version 1.2.1), and molecular fingerprints were generated using RDKit (version 2019.09.03).

Accuracy and the coefficient of determination (r2) were used to compare the models. The accuracy is defined as:

$$\text{Accuracy} = \frac{\text{TP} + \text{TN}}{\text{TP} + \text{TN} + \text{FP} + \text{FN}}$$

where TP (true positives) and TN (true negatives) denote correctly predicted positive and negative samples, respectively, and FP (false positives) and FN (false negatives) denote incorrectly predicted samples.

The R^2 is defined as:

$$R^2 = 1 - \frac{\left(\Sigma_{i=1}^{n}(y_i - \hat{y}_i)^2\right)}{\left(\Sigma_{i=1}^{n}(y_i - \bar{y})^2\right)}$$

where y_i represents the observed values, $\hat{y}_i$ the predicted values, and $\bar{y}$ the mean of the observed values.

Extended content S5. Case study of key sub-sectors and specific chemicals in the U.S. market

To analyze key sub-sectors and specific chemicals in the U.S. market, the chemical production volume data for 2016–2019 were obtained from the U.S. Environmental Protection Agency's Chemical Data Reporting dataset[24]. To ensure a clearer and more meaningful presentation of results, related industrial sectors were combined. For instance, chemicals used in "synthetic dye and pigment manufacturing" and "paint and coating manufacturing" were grouped under "pigment and coating manufacturing." A detailed grouping of these sectors is provided in **Table S5**. For the total emissions analysis, the median carbon footprint of all the high-carbon-footprint chemicals collected from LCA databases and chemical producers (17.27 kg CO_2-eq/kg) was used as a proxy for the high-carbon-footprint chemicals.

Over 3700 chemicals with sector information were each classified as either high-carbon-footprint chemicals (above 10 kg CO_2-eq/kg) or chemicals with carbon footprints of <= 10 kg CO_2-eq/kg. For 1600 chemicals, sector-specific usage volume data were available, and the minimum total emissions of each sector could be

quantified. Confidential chemicals, recycled chemicals, and chemicals without known volumes were excluded from the analysis. Since a single chemical—such as urea—can serve as a raw material across multiple industrial sectors (e.g., agricultural chemicals, plastics, and resins), we only included sector-specific usage data when companies explicitly reported the use of a given chemical within a particular sector, as recorded in the U.S. Environmental Protection Agency's Chemical Data Reporting database.

To showcase the use of FineChemDB in analyzing chemical carbon footprints across industrial sectors, or within specific jurisdiction, and informing sustainable transition strategies, we integrate FineChemDB data with the 2016–2019 U.S. Chemical Data Reporting dataset[24].

The U.S. Chemical Data Reporting dataset includes detailed information on the industrial sectors that use specific chemicals as input raw materials, including petrochemical manufacturing, plastics and rubber production, and agricultural chemical manufacturing, along with use volumes. We estimate the carbon footprint of over 3,700 chemicals used in manufacturing products for 13 sectors (for the list of sectors, see **Table S6**). Notably, an additional ~1,000 organic chemicals were reported by companies but were either confidential or outside the applicability domain of FineChem2. These chemicals were mostly specialized chemicals with low reported production or use volumes and together accounted for 7% of the total reported mass of organic chemicals in the dataset. This suggests that their influence on mass-weighted sectoral analyses is likely limited. However, because their carbon footprints are unknown, the reported sectoral carbon footprints should be interpreted as minimum estimates, as some excluded chemicals may have disproportionately high carbon footprints. Comparisons between individual sectors should also be avoided because of unavoidable data gaps, including confidential use volumes, chemicals in imported products, and the complexity of chemical uses across sectors.

Overall, the proportion of high-carbon-footprint chemicals (by number of chemicals) used as inputs across most industrial sectors remains below 10%, with the exception of plastics and rubber, food products, paper, and pigment and coating manufacturing, which exhibit notably higher shares—21%, 13%, 14%, and 11%, respectively. In contrast, raw chemical materials utilized in petrochemicals, adhesives, and construction generally exhibit lower carbon footprints.

While the mass share (usage volume) of high-carbon-footprint chemicals is generally modest (typically below 5%), their contribution to total greenhouse gas emissions can be disproportionately large. For instance, in the paper manufacturing, construction, and plastics and rubber sectors, high-carbon-footprint chemicals account for just 5%, 4%, and 1% of the total chemical use by volume, yet contribute 20%, 17%, and 6% of estimated carbon emissions from chemical inputs in each sector. These findings indicate that even small quantities of high-carbon-footprint chemicals can significantly impact overall carbon emissions.

This highlights the importance of targeted substitution and mitigation strategies, where replacing high-emission chemicals—such as specific additives or dyes—can lead to substantial reductions in embedded greenhouse gas emissions.

Interestingly, chemicals used in plastics and rubber manufacturing have both high volumes and carbon footprints. The reason behind this is the combination of bulk chemical inputs and complex chemical additives, such as various plasticizers. This is also evident from the proportion of high-carbon-footprint chemicals (over 21% by number). Despite the substantial carbon footprints of these chemical additives, their environmental impacts are often overlooked in previous studies due to the lack of detailed life cycle inventory data[25,26]. Such simplifications may lead to misleading conclusions and a systematic underestimation of the true environmental footprint of final plastic products. Future studies on the contributions of chemical additives to the overall carbon footprint of plastics are warranted.

Extended content S6. Prediction of the carbon footprint of chemical mixtures.

To extend FineChem2's prediction capabilities to chemical mixtures—particularly those that include inorganic ions such as salts—we developed an integrated pipeline that combines model-based and proxy-based estimation strategies. Specifically, the organic component of the mixture is evaluated using the FineChem2 model, while the inorganic component is estimated using a predefined proxy list constructed from available life cycle inventory data.

This proxy list captures common inorganic ions (e.g., Na^{+}, Cl^{-}, SO_4^{2-}, PO_4^{3-}) and includes estimated carbon footprints derived from typical industrial synthesis routes. For example, when sodium ions are introduced into a compound, they may originate from various industrial reactants such as sodium hydroxide, calcium oxide, or potassium hydroxide, each carrying different upstream emissions. To reflect this variability, we estimate the carbon footprint contribution of the sodium ion based on the associated reactants and include an uncertainty range that accounts for the choice of reactants (**Table S1**). This approach allows us to provide a more realistic and flexible estimate of the inorganic component's carbon footprint.

Each component of the mixture—organic and inorganic—is evaluated independently using the respective method, and their carbon footprints are then aggregated proportionally based on mass contribution to yield the final carbon footprint of the chemical mixture. For example, for a 1 kg chemical mixture composed of two organic components (together accounting for 0.9 kg) and an inorganic component (0.1 kg) that can be introduced from one source chemical, the overall carbon footprint is calculated using the following weighted average formula:

$$PCF_{chemical\ mixture} = \frac{0.9 \times (PCF_{organic\ component\ 1} \times Mass_{organic\ component\ 1} + PCF_{organic\ component\ 2} \times Mass_{organic\ component\ 2})}{Mass_{organic\ component\ 1} + Mass_{organic\ component\ 2}} + \frac{0.1 \times PCF_{ionic\ component}}{Num.\ of\ inorgnaic\ component\ introduced\ by\ the\ source\ chemical}$$

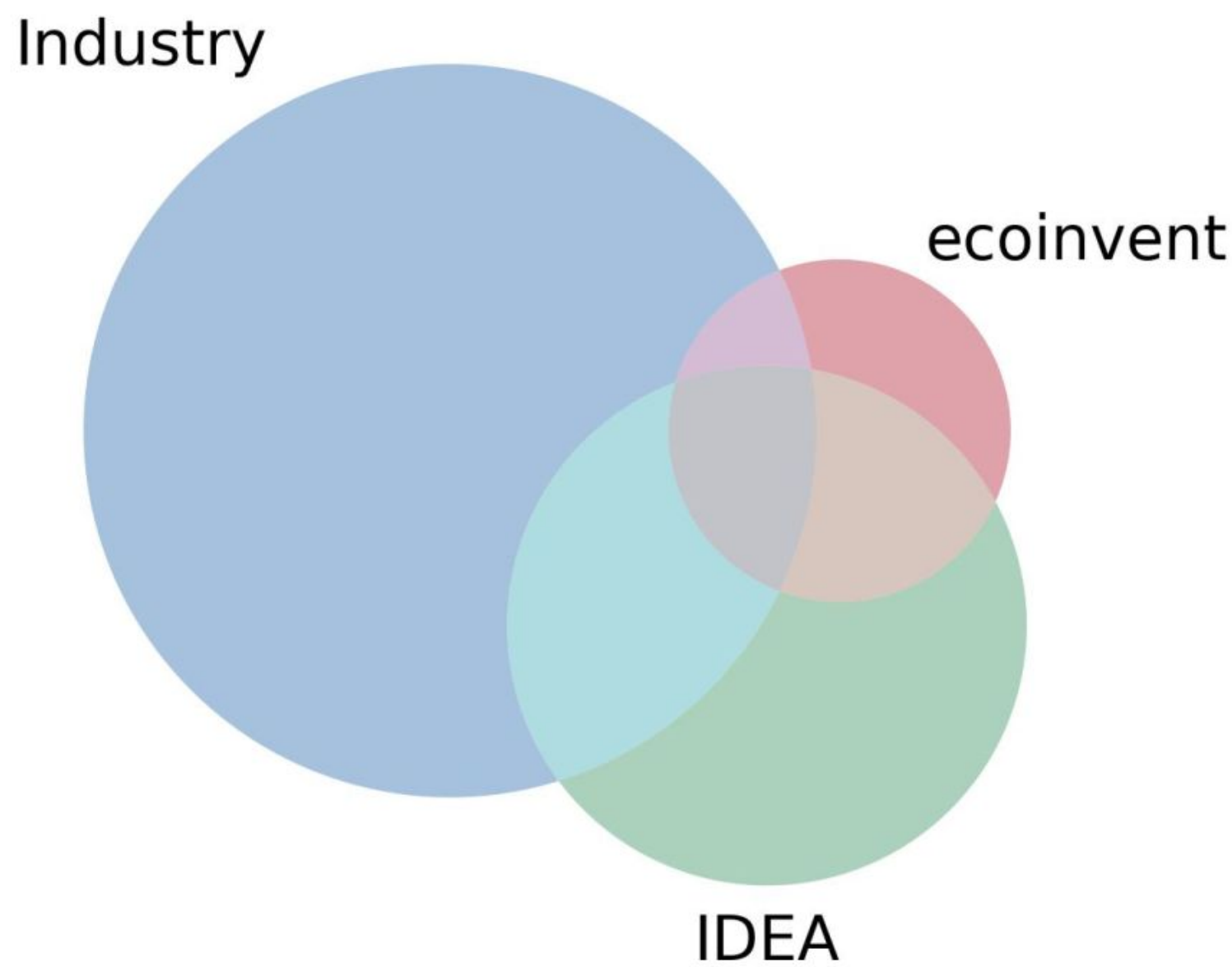

Figure S1. The Venn plot of chemicals collected from the chemical industry, IDEA v2.3, and ecoinvent v3.8. For ecoinvent, datasets contaminated with direct proxy data or with major proxy use in their educts were excluded, and only high-quality datasets were included for modeling. The industry section includes first-hand chemical carbon footprint data from the industry and from a previous study of 20 chemicals[27].

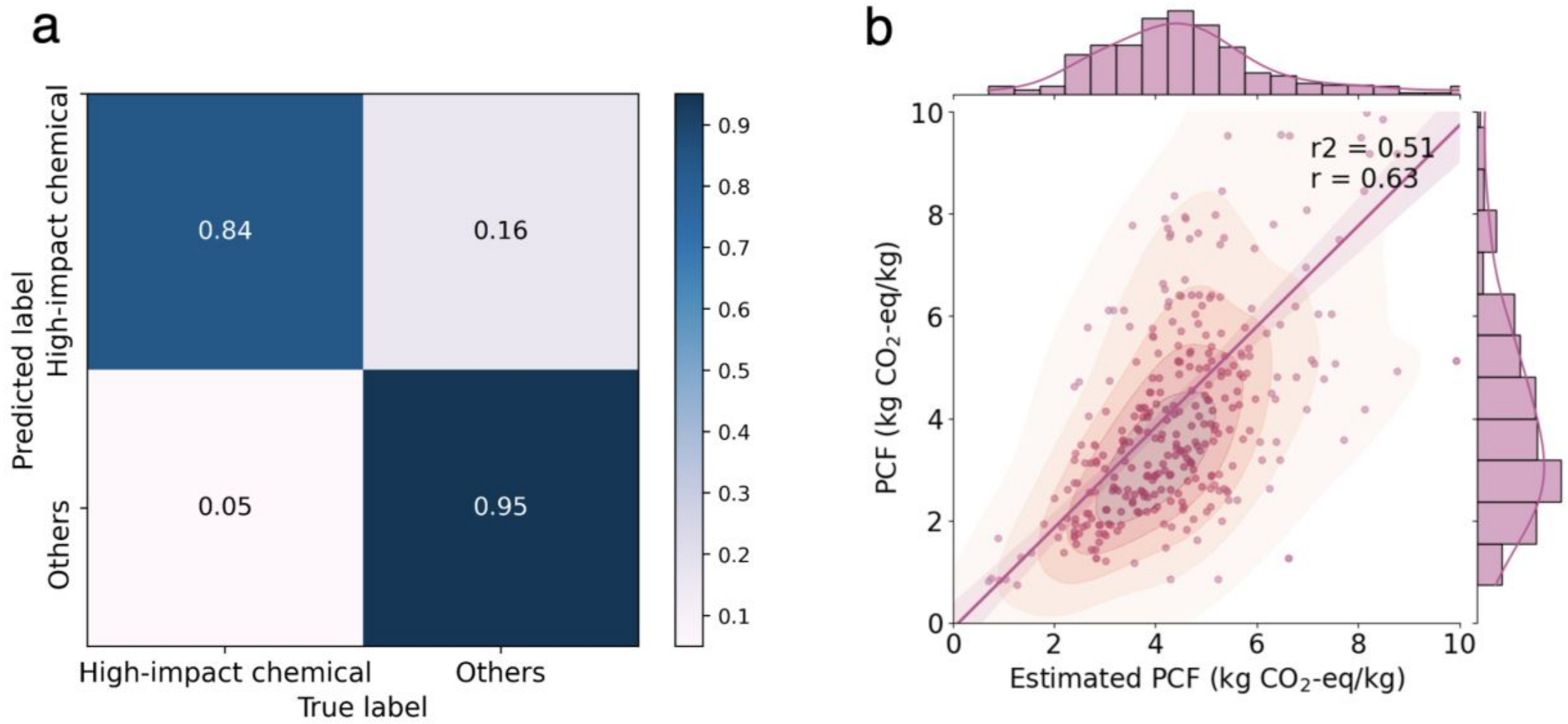

Figure S2. Evaluation of the updated prediction models. (a) The confusion matrix displays the classification model's accuracy in distinguishing between high and low-moderate carbon footprint chemicals. (b) The correlation between the estimated and true carbon footprint of chemicals.

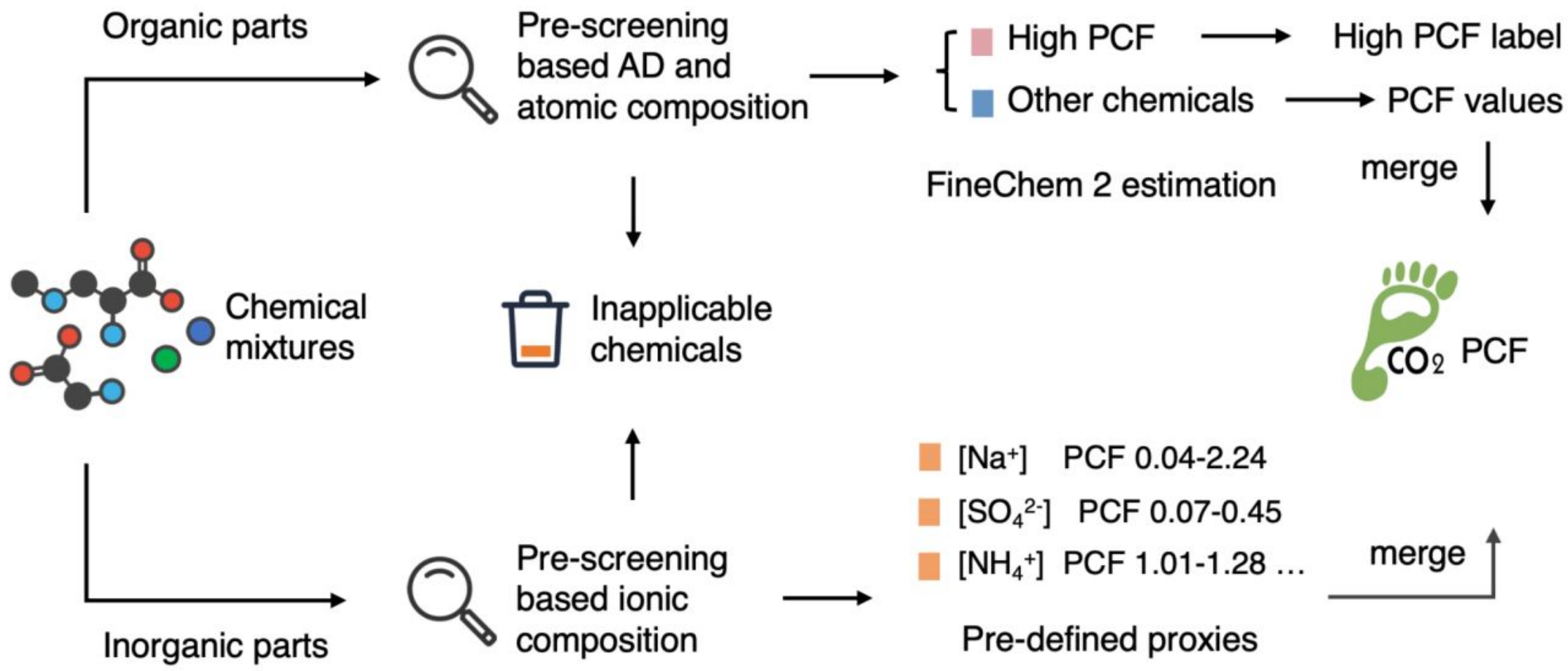

Figure S3. Framework for estimating chemicals' carbon footprints and model evaluation. The framework for estimating chemicals' carbon footprints. The estimation process begins with a pre-screening of the organic components in each chemical based on the classification model's applicability domain and atomic composition. Following this, a classification model categorizes chemicals into high-PCF (> 10 kg CO_2-eq/kg) and low-moderate PCF (≤ 10 kg CO_2-eq/kg) groups. Chemicals classified as low-moderate PCF proceed to a regression model for precise PCF estimation. For permissible inorganic components, PCF values are retrieved from predefined proxies and integrated into the overall chemical footprint.

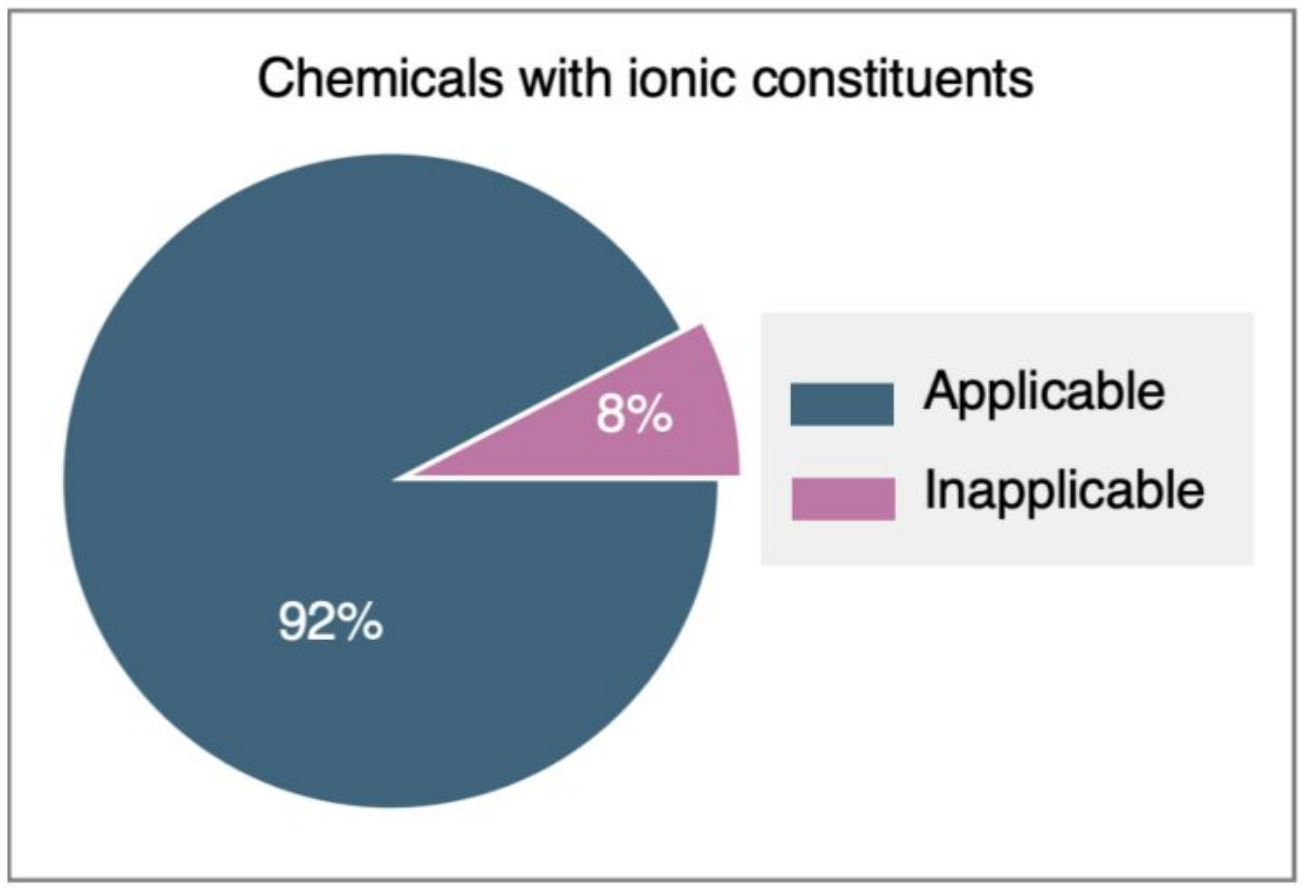

Figure S4. Proportion of chemicals with ionic constituents that are within the model's applicability domain versus those that are not.

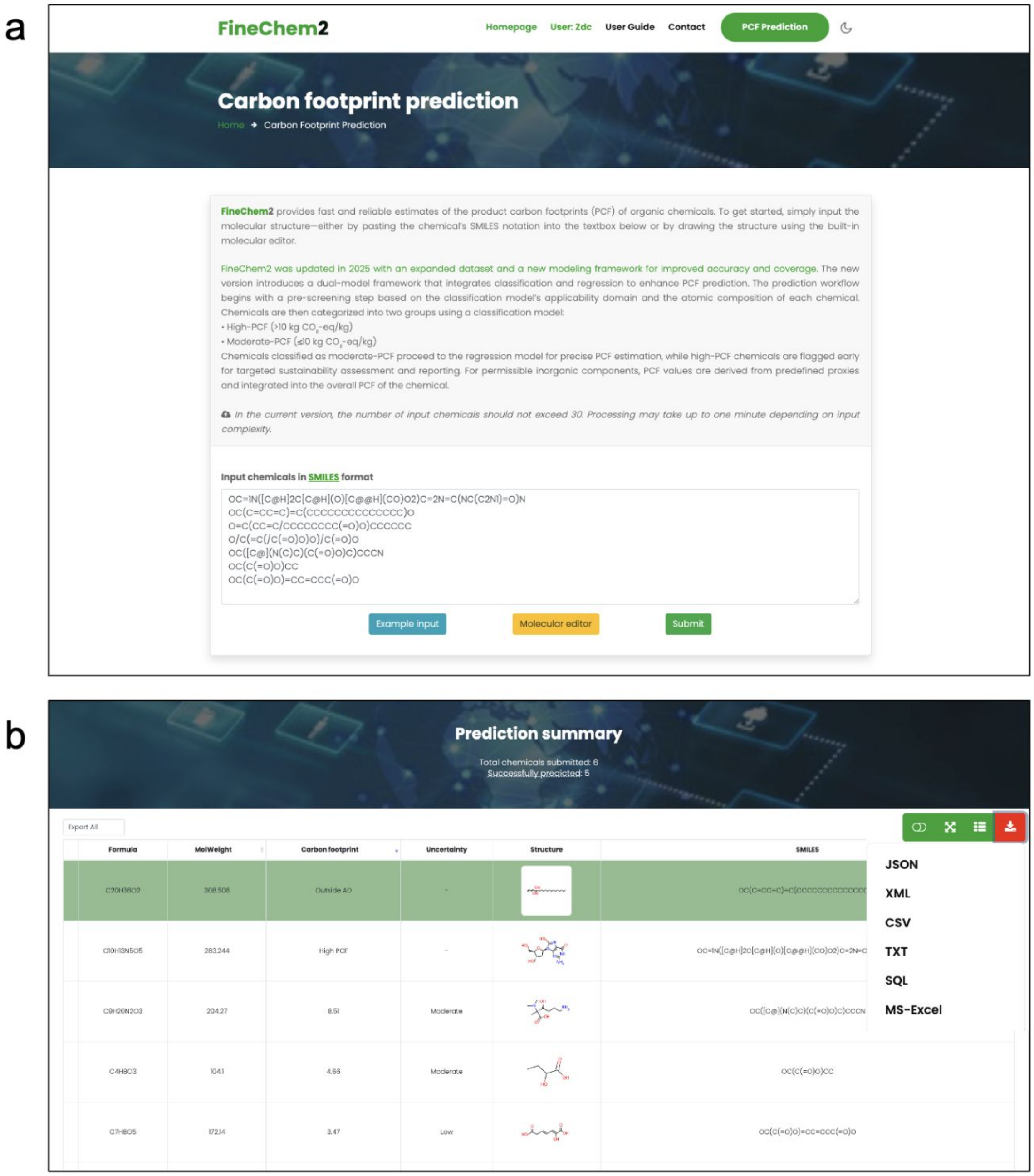

Figure S5. Interface of FineChemDB powered by FineChem2.1 prediction models. (a) Search interface: Users can search for chemicals by molecular structure using the embedded search bar or by drawing chemical structures directly within the interface. The platform supports both single-compound queries and batch uploads, making it suitable for high-throughput screening. (b) Prediction result interface: Once a chemical is selected, the tool displays its predicted carbon footprint, within/outside applicability domain, classification category (e.g., low-moderate or high carbon footprints), and uncertainty ranges (e.g., low, moderate, and high) of the prediction.

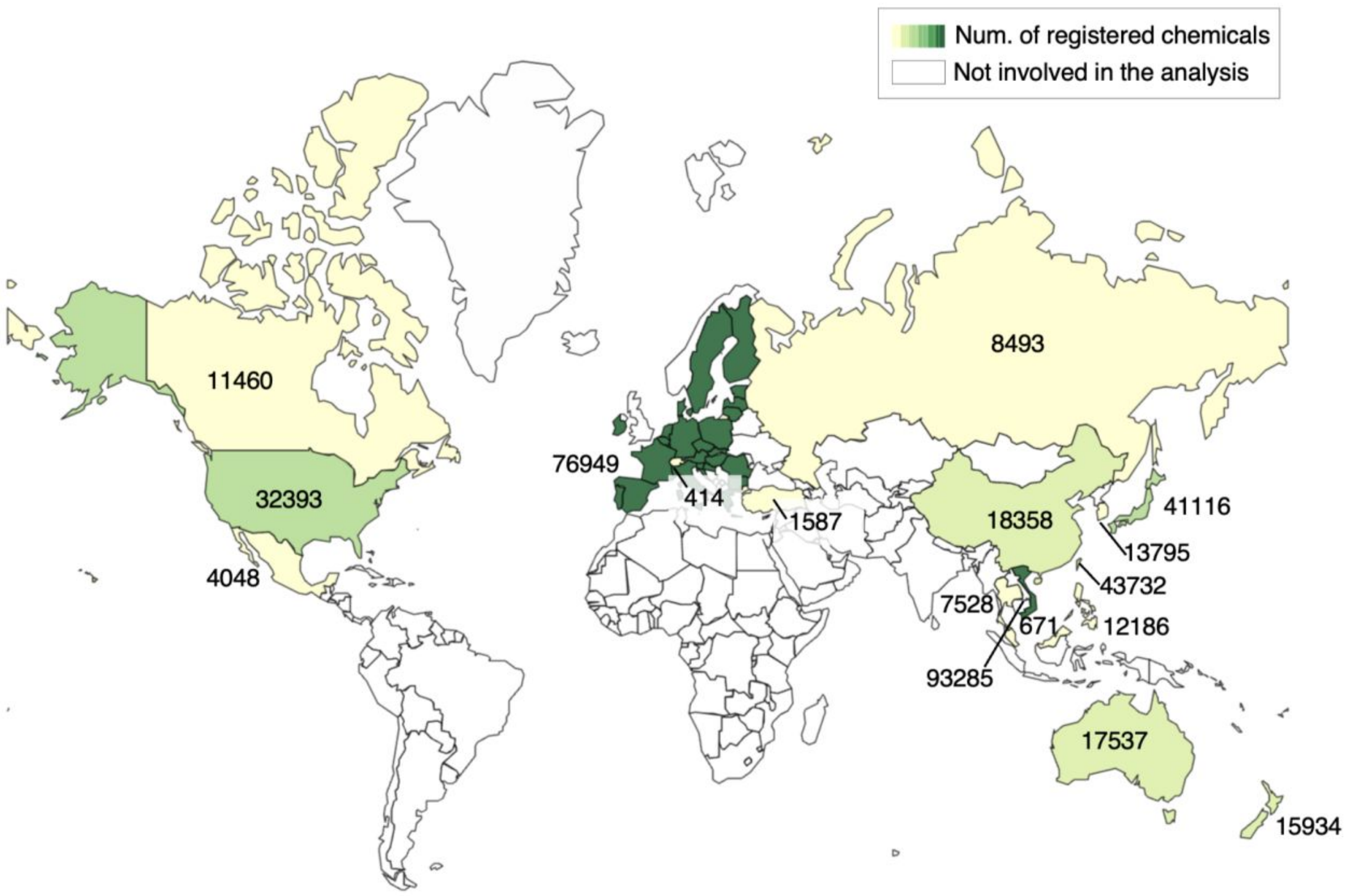

Figure S6. Number of unique organic chemicals registered in various countries or regions.

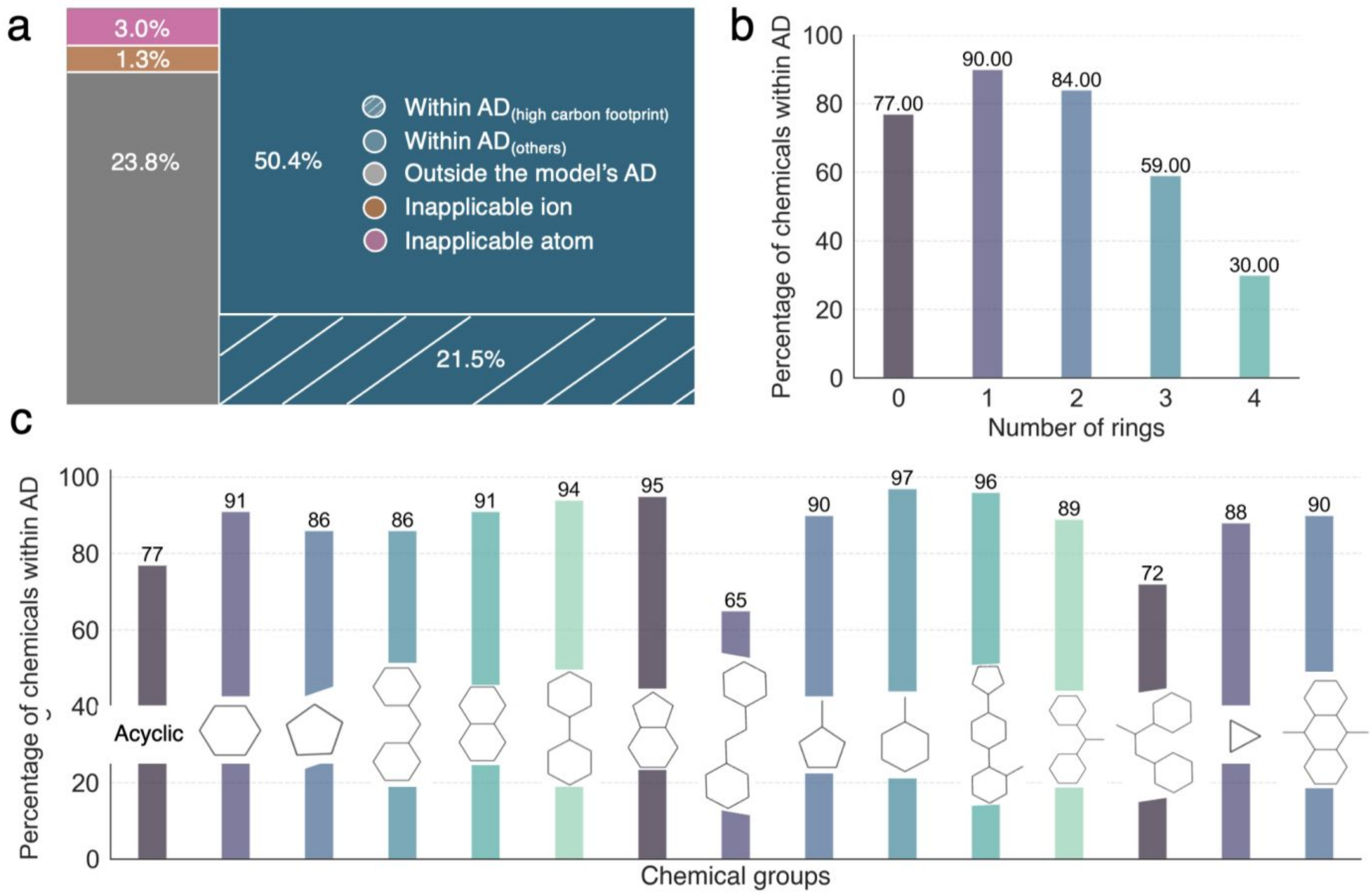

Figure S7. Analysis of the model's applicability domain. (a) Proportion of chemicals that fall within versus outside the model's applicability domain. The remaining chemicals fall outside the AD due to three primary reasons: (i) structural distance beyond the model's training chemical space, (ii) presence of inorganic ions not covered by the proxy list, or (iii) inclusion of atoms not represented in the training dataset. Specifically, chemicals excluded due to inapplicable atomic compositions or inorganic ions represent a relatively small fraction compared to those excluded based on structural distance criteria. Ratio of different groups of chemicals within the model's applicability domain based on the number of rings (b) and common types of molecular frameworks (c).

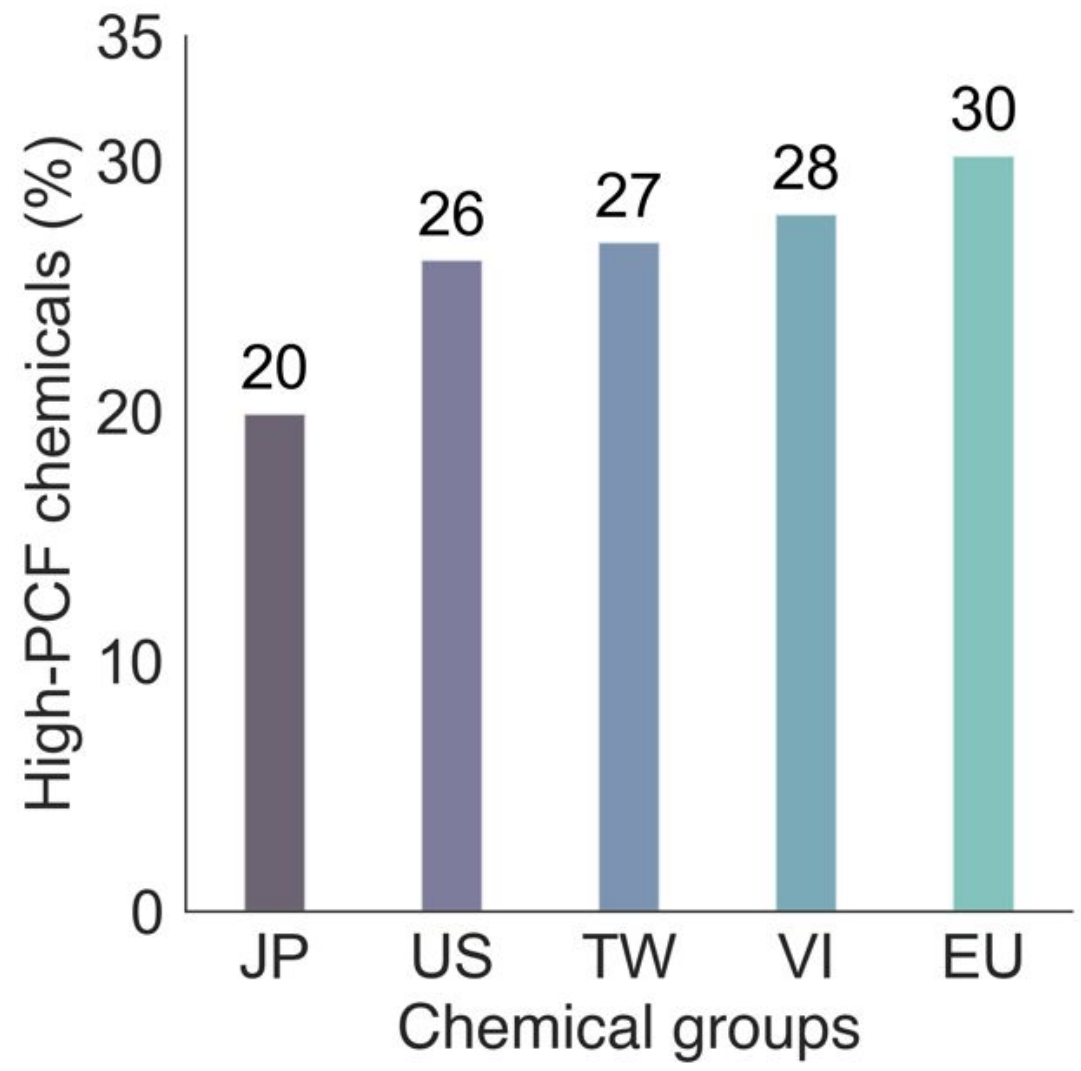

Figure S8. Comparisons of the share of high-carbon-footprint chemicals registered in various countries and regions. These differences should not be interpreted as reflecting country-specific production carbon footprints, especially since production volumes were not available. FineChemDB provides structure-based carbon footprint estimates, and the observed differences reflect variations in inventory coverage, regulatory systems, and the composition of chemicals registered in different jurisdictions, rather than differences in regional production routes or electricity mixes.

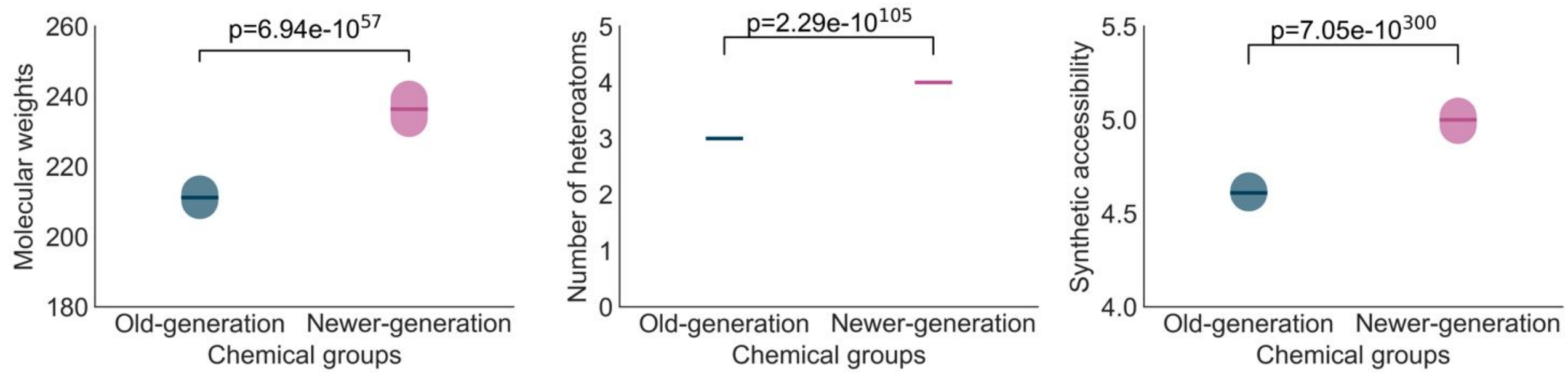

Figure S9. Comparisons of molecular weights, number of heteroatoms, and synthetic accessibility of existing and newly registered chemicals. The lines indicate the median carbon footprint values of existing and newly registered chemicals. The ovals indicate the 95% confidence intervals of all analyzed chemicals, calculated by taking a percentile interval of the *bootstrap distribution* via the Seaborn package. The lines in the ovals refer to the median values.

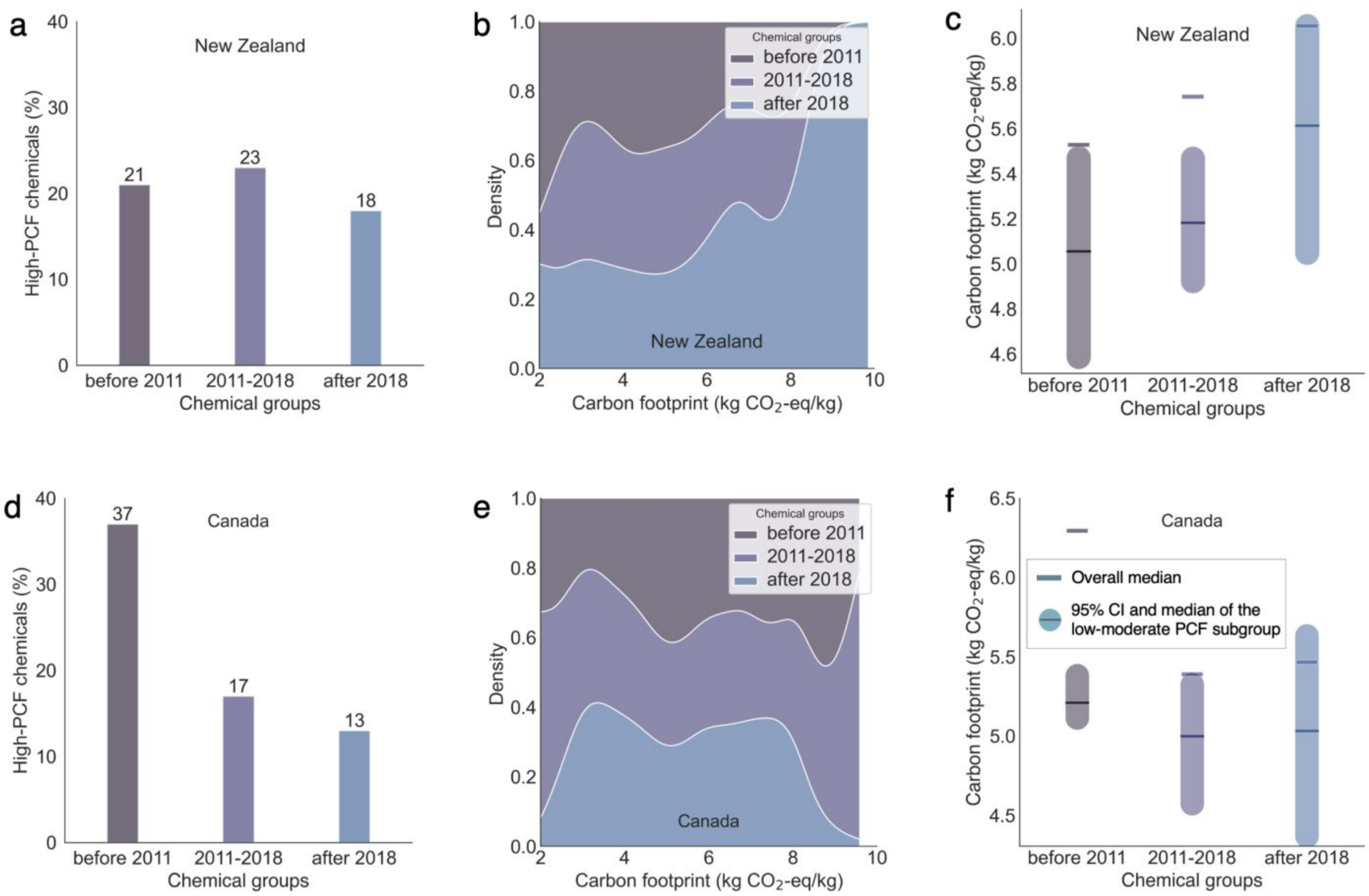

Figure S10. The trends of the carbon footprint of chemicals registered in New Zealand and Canada. (a) Comparisons of the ratio of high-PCF chemicals registered in different time periods in New Zealand. (b) The normalized stacked distribution of the PCF values for chemicals with a low-moderate carbon footprint (≤ 10 kg CO_2-eq/kg) registered in different time periods in New Zealand. (c) Comparisons of the PCF values of chemicals with a low-moderate carbon footprint registered in different time periods in New Zealand. (d) Comparisons of the ratio of high-PCF chemicals registered in different time periods in Canada. (e) The normalized stacked distribution of the PCF values for chemicals with a low-moderate carbon footprint registered in different time periods in Canada. (f) Comparisons of the PCF values of chemicals registered in different time periods in Canada. The lines out of the ovals in (c) and (f) indicate the median PCF of all chemicals within each group, including both low-moderate and high-PCF chemicals. The ovals in (c) and (f) indicate the 95% confidence intervals of the low-moderate PCF chemicals (≤ 10 kg CO_2-eq/kg), calculated by taking a percentile interval of the *bootstrap distribution* via the Seaborn package. The lines in the ovals refer to the median PCF of low-moderate PCF chemicals. This analysis focuses solely on the newer-generation chemicals, excluding those that were already present in the databases at the time of their release.

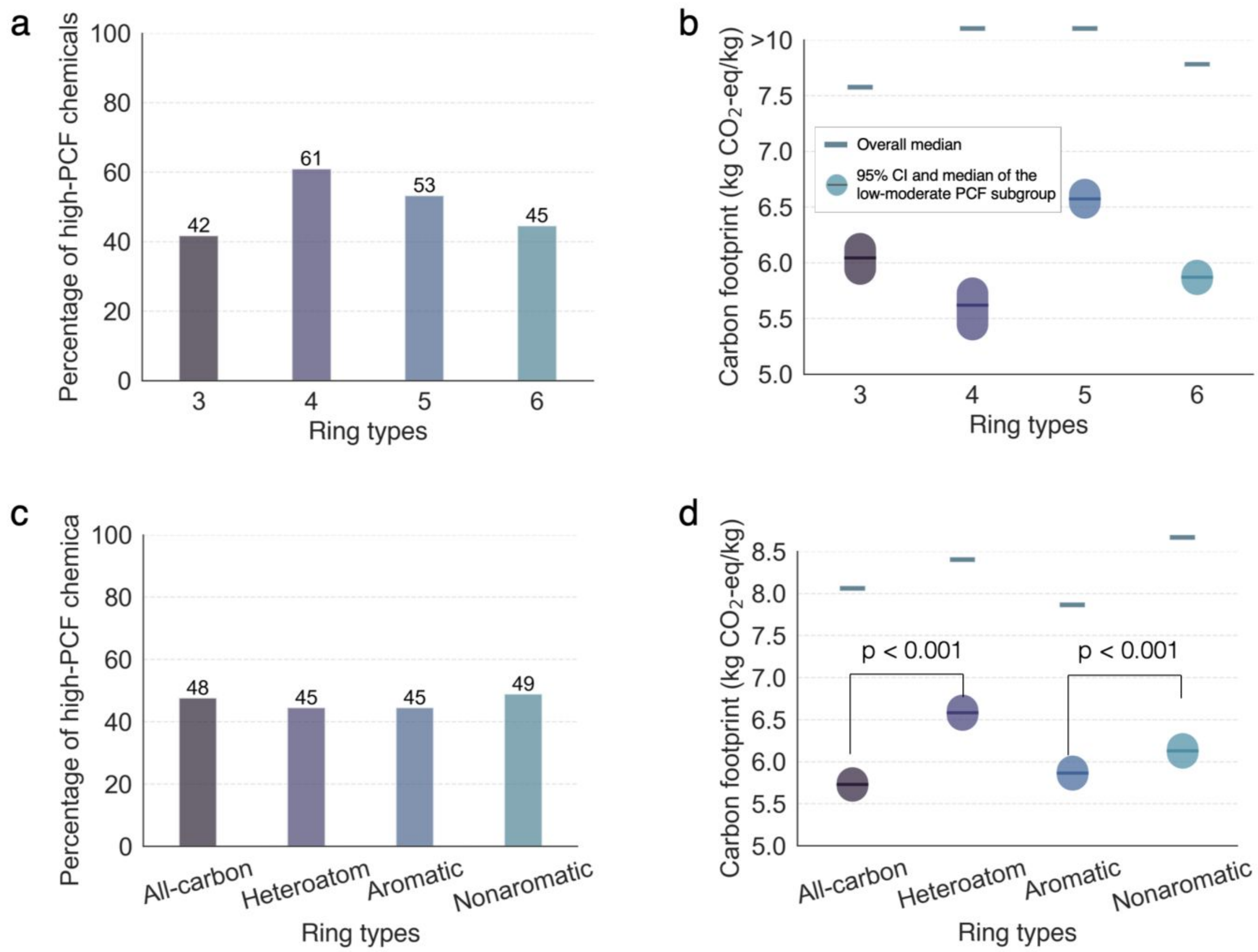

Figure S11. Comparisons of the carbon footprint of chemicals with different ring types. (a) Comparison of the proportion of high-PCF chemicals within different types of rings, including three-member rings, four-member rings, five-member rings, and six-member rings. (b) Comparisons of the PCF values of chemicals with different types of rings. (c) Comparison of the proportion of high-PCF chemicals within different types of rings. All-carbon ring: ring composed entirely of carbon atoms. Heteroatom ring: ring that contains at least one non-carbon atom. Aromatic ring: ring whose atoms are all marked aromatic by RDKit. Nonaromatic ring: ring that is not fully aromatic. (d) Comparisons of the PCF values of chemicals with different types of rings. The lines in (b) and (d) indicate the median PCF of all chemicals within each group, including both low-moderate and high-PCF chemicals. The ovals indicate 95% confidence intervals of the low-moderate PCF chemicals (≤ 10 kg CO_2-eq/kg), calculated by taking a percentile interval of the *bootstrap distribution* via the Seaborn package. The lines in the ovals refer to the median PCFs of low-moderate PCF chemicals.

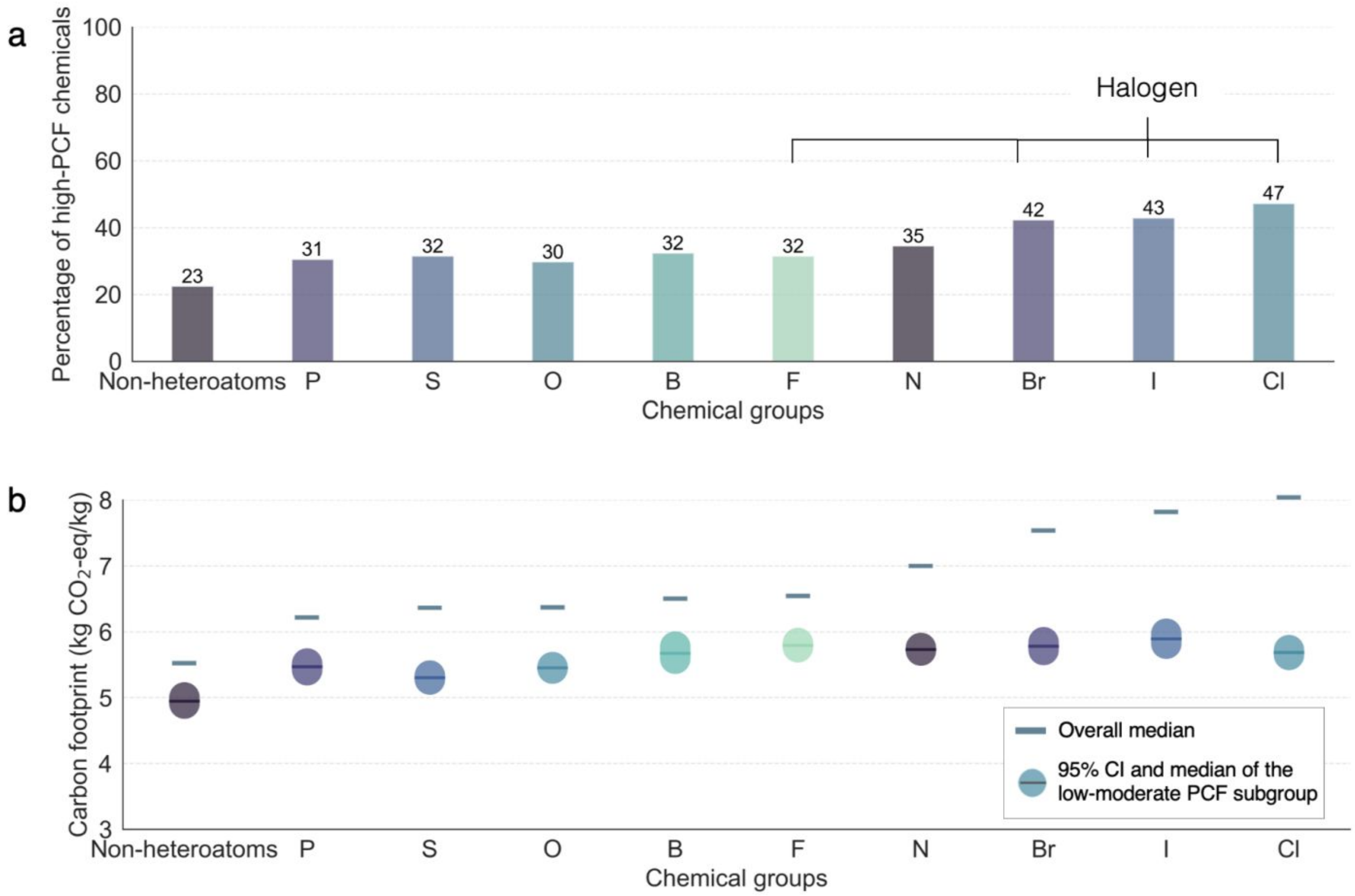

Figure S12. Comparisons of the carbon footprint of chemicals with different element compositions. For instance, the N group includes chemicals with at least one nitrogen atom involved. The non-heteroatoms group includes chemicals that consist only of carbon and hydrogen atoms. (a) Comparison of the proportion of high-PCF chemicals within different groups. (b) Comparisons of the PCF values of chemicals with different element compositions. A chemical containing multiple elements is assigned to multiple groups. Additionally, we include a category for chemicals without any heteroatoms, comprising only carbon and hydrogen. The lines indicate the median PCF of all chemicals within each group, including both low-moderate and high-PCF chemicals. The ovals indicate 95% confidence intervals of the low-moderate PCF chemicals (≤ 10 kg CO_2-eq/kg), calculated by taking a percentile interval of the *bootstrap distribution* via the Seaborn package. The lines in the ovals refer to the median PCFs of low-moderate PCF chemicals.

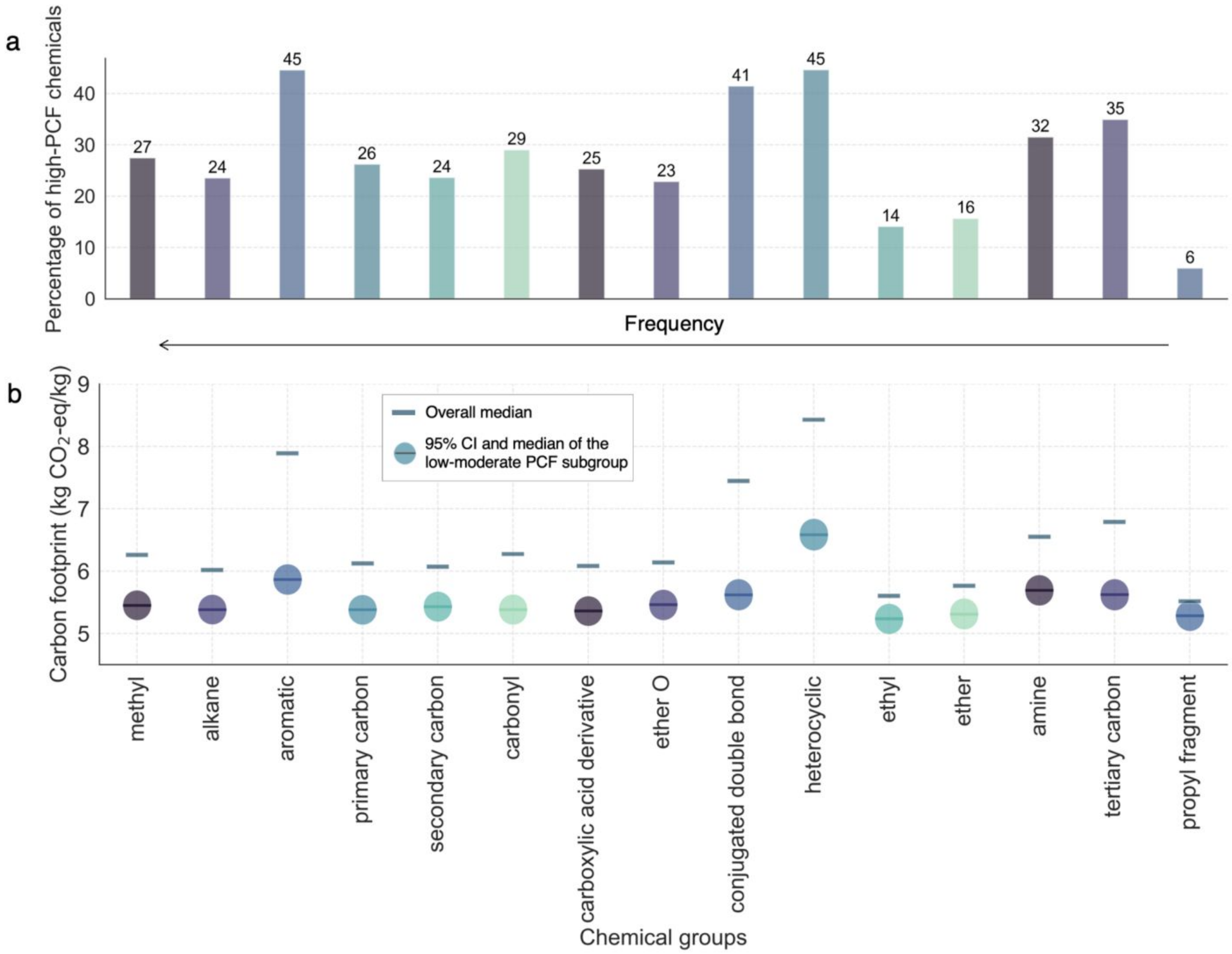

Figure S13. Comparisons of the carbon footprint of chemicals with the top 15 common functional groups. For instance, the 'methyl' group includes chemicals containing at least one methyl group. (a) Comparison of the proportion of high-PCF chemicals within different groups. (b) Comparisons of the PCF values of chemicals with different functional groups. The lines indicate the median PCF of all chemicals within each group, including both low-moderate and high-PCF chemicals. The ovals indicate 95% confidence intervals of the low-moderate PCF chemicals (≤ 10 kg CO_2-eq/kg), calculated by taking a percentile interval of the *bootstrap distribution* via the Seaborn package. The lines in the ovals refer to the median PCFs of low-moderate PCF chemicals. Functional groups of chemicals were analyzed using the exmol toolkit (https://ur-whitelab.github.io/exmol/).

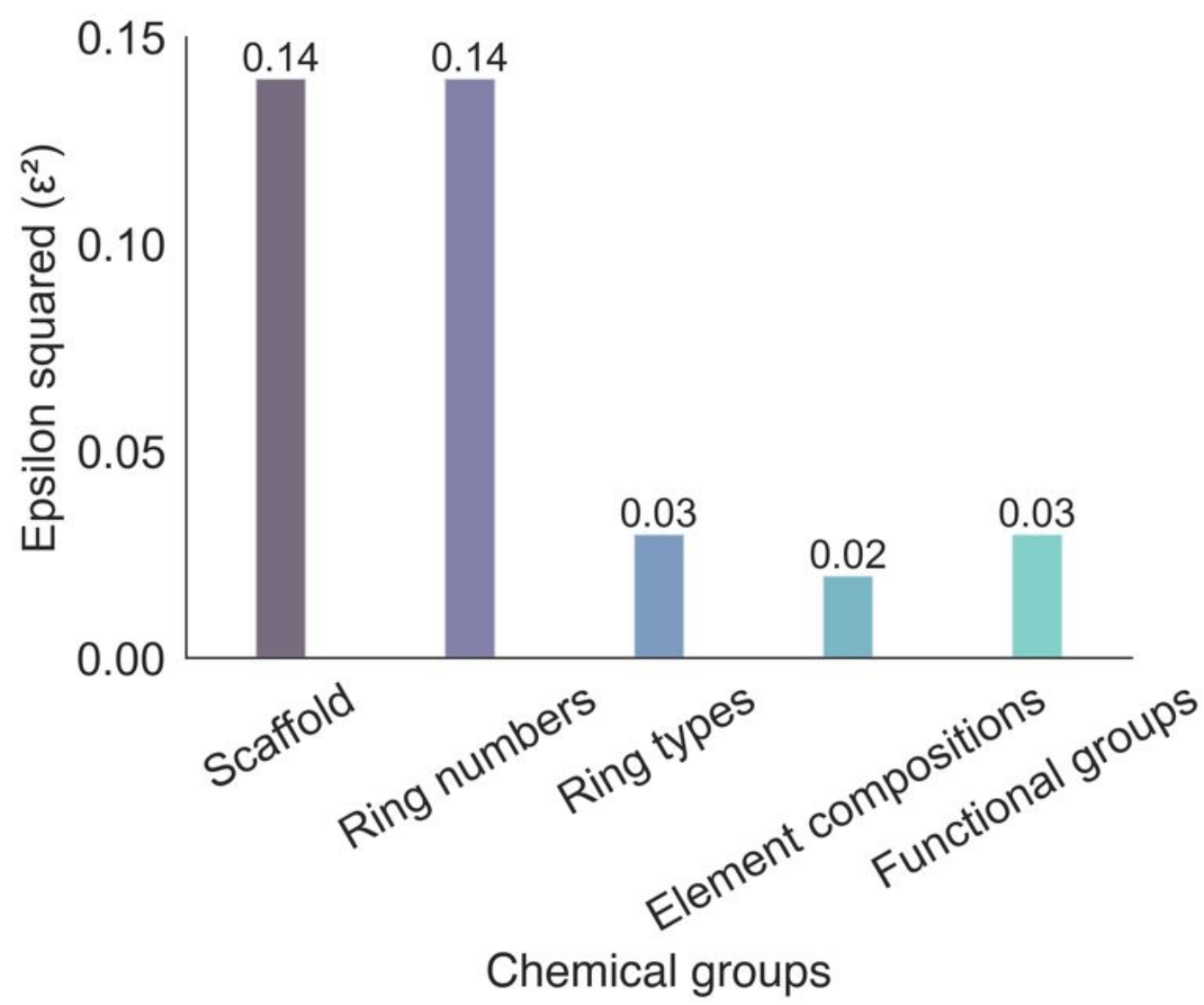

Figure S14. Comparisons of the epsilon squared (ε^2) of Kruskal-Wallis H-statistic values of various molecular characteristics. The epsilon squared (ε^2) of Kruskal-Wallis H-statistic values presents a statistically significant difference among all categories combined. The higher the value, the more significant the overall difference. Molecular scaffolds and elemental compositions were defined following RDKit protocols. Specifically, molecular scaffolds were generated by removing all side chains while retaining ring systems and linkers required to connect multiple rings (i.e., Murcko scaffolds). During this process, heteroatoms in the scaffold were converted to carbon atoms, and all bond orders were reduced to single bonds to standardize structural representation. Additionally, the first atom of any exocyclic substituent was retained, in accordance with RDKit recommendations.

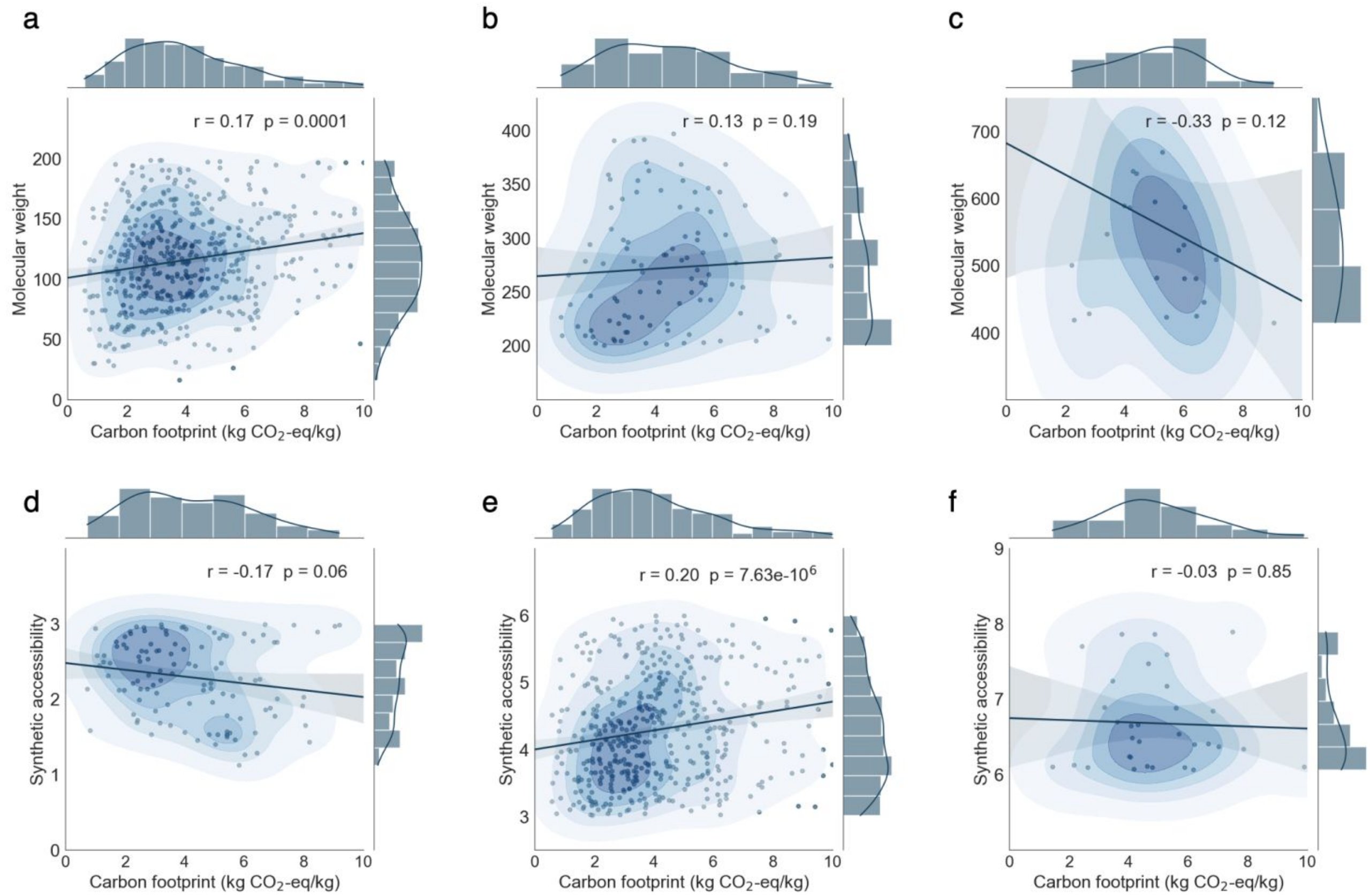

Figure S15. Correlations between chemical carbon footprints and selected indicators. (a-c) The correlation between carbon footprints and molecular weights for chemicals with different ranges (0-200Da, 200-400Da, and 400-700 Da) of molecular weights. (d-f) The relationship between carbon footprints and synthetic accessibility for chemicals with different ranges (0-3, 3-6, and 6-9) of synthetic accessibility. The r value presents the Spearman correlation coefficient.

Table S1. Proxies to present the contribution of inorganic compositions to the carbon footprint of chemicals.

SMILES	Carbon footprints	Collected source chemicals with high quality life cycle inventory data
[NH4+] / N	1.09-1.49	NH_4Cl, NH_4HCO_3, $(NH_4)_2SO_4$, NH_4PO_4, NH_4NO_3
[Cl-]/ Cl	0.04-2.73	NH_4Cl, NaCl, KCl, $CaCl_2$, $BaCl_2$, $FeCl_2$, $FeCl_3$
[F-]/ F	3.493	F_2
[Br-]/ Br	4.2	Br_2
[I-]/ I	2.8	I_2
OP(=O)(O)O	1.98-3.28	H_3PO_4
[O-]S(=O)(=O)[O-]/ OS(=O)(=O)O	0.07-0.45	$(NH_4)_2SO_4$, K_2SO_4, $BaSO_4$, $FeSO_4$, Al_2(SO4)$_3$, $ZnSO_4$, $CuSO_4$,
[N+](=O)(O)[O-]	0.99-1.52	$NaNO_3$, KNO_3
[OH-]	0.82-2.73	KOH, NaOH, LiOH, $Mg(OH)_2$,
[Li+]	1.05-5.81	LiCl, LiOH, Li_2CO_3
[Na+]	0.041-1.12	NaOH, NaCl, Na_2CO_3, $NaHCO_3$, $NaNO_3$
[Mg+2]	1.63-5.31	$Mg(OH)_2$, $MgCO_3$
[K+]	0.52-2.73	KOH, KCl, K_2CO_3, K_2SO_4, KNO_3
[Ca+2]	0.53-2.02	$CaCl_2$, $Ca_3(PO_4)_2$
[Ba+2]	1.43-7.87	$BaCl_2$, $BaCO_3$, $BaSO_4$
[Fe+2]	0.25-0.28	$FeCl_2$, $FeSO_4$
[Fe+3]	0.43	$FeCl_3$
[Al+3]	0.37	Al2(SO4)$_3$
[Zn+2]	0.75-1.84	$ZnSO_4$, [Zn]=O
[Cu+2]	3.79	$CuSO_4$
[Ag+]	85.96	$AgNO_3$

Table S2. The number of chemicals involved in various national chemical inventories.

Note: "Personal correspondence (PC)" indicates that the data was obtained through direct communication.

Jurisdiction	Inventories	Entries	Num. of unique organic chemicals	Ref	First release time
Australia	Australian Industrial Chemical Inventory	39594	17537	1	1999
Canada	Canada Domestic Substances List<br>Canada Pesticide Product Information Database	29905	11460	2, 3	1994<br>2017
China	Inventory of Existing Chemical Substances	46803	18358	PC	
EU	ECHA Pre-registered Substances List<br>EU REACH Regulation<br>EU Cosmetic ingredient database<br>EU Public Health - Union Register of Medicinal Products	181183	76949	4-8	2008<br>2006<br>2022<br>-
Japan	NITE Lists of Chemical Substances<br>Japanese Industrial Safety and Health Act	118719	41116	9, 10	1973<br>1972
Malaysia	Malaysian Environmentally Hazardous Substances System<br>Register of Potentially Hazardous Chemical and Biological Substances	1472	671	11, PC	2023<br>-
Mexico	National Inventory of Chemical Substances	5852	4048	PC	-
New Zealand	Inventory of Chemicals	29118	15934	12	2006
Nordic countries	Substances in Preparation	31738	12940	13	2000
Philippines	Inventory of Chemicals and Chemical Substances	22277	12186	14	1995
Russia	Register of Potentially Hazardous Chemical and Biological Substances	12914	8493	PC	-
South Korea	Existing Chemicals List	46234	13795	15	1996
Switzerland	New Substance Registration	2242	414	16	2021
Taiwan	Chemical Substance Inventory	104049	43732	17	2015
Thailand	Existing Chemicals Inventory v1 (2012.34)	18691	7528	PC	2012

	Existing Chemicals Inventory v2 (2016.34)				2016
Turkey	Chemical Substance Inventory	2889	1587	PC	-
United States	Toxic Substance Control Act<br>Active Pesticide Product Registration Informational Listing	88832	32393	18, 19	1976<br>-
Vietnam	National Chemical Inventory	175335	93285	20	2016
Total		888,348		131,765 (after removal of duplicates)	

Table S3. Coefficient of determination (r^2) of the FineChem2 regression and baseline ML models.

ML models	r^2
FineChem 2 model	0.51±0.133
ECFP4-SVM	0.288±0.066
RDKIT-RF	0.31±0.073
ECFP4-RF	0.355±0.183
RDKIT-ANN	0.121±0.09
MACCS-RF	0.302±0.06
MACCS-ANN	0.174±0.069
MACCS-SVM	0.169±0.048
ECFP4-ANN	-0.096±0.357
RDKIT-SVM	0.223±0.086

Table S4. Accuracy of the FineChem2 classification and baseline ML models.

ML models	Accuracy
FineChem 2 model	0.93±0.049
ECFP4-RF	0.914±0.027
RDKIT-ANN	0.914±0.027
ECFP4-SVM	0.911±0.038
MACCS-RF	0.911±0.035
MACCS-SVM	0.905±0.031
MACCS-ANN	0.898±0.044
RDKIT-RF	0.895±0.028
ECFP4-ANN	0.891±0.019
RDKIT-SVM	0.875±0.03

48 **Table S5. Consolidation of related industrial sectors in the 2016-2019 U.S. Chemical Data**
49 **Reporting dataset for better clarity.**

New sector names	Original sector names
Others	All other petroleum and coal products manufacturing<br>All other chemical product and preparation manufacturing<br>All other basic organic chemical manufacturing<br>All other basic inorganic chemical manufacturing<br>Other (requires additional information)
Construction	Construction
Petrochemical manufacturing	Petrochemical manufacturing
Printing chemical manufacturing	Printing and related support activities<br>Printing ink manufacturing
Adhesive manufacturing	Adhesive manufacturing
Plastics and rubber manufacturing	Plastics product manufacturing<br>Plastics material and resin manufacturing<br>Rubber product manufacturing<br>Synthetic rubber manufacturing
Agricultural chemical manufacturing	Pesticide, fertilizer, and other agricultural chemical manufacturing
Cleaning chemical manufacturing	Soap, cleaning compound, and toilet preparation manufacturing
Pigment and coating manufacturing	Synthetic dye and pigment manufacturing<br>Paint and coating manufacturing
Food product manufacturing	Food, beverage, and tobacco product manufacturing
Electronic product manufacturing	Computer and electronic product manufacturing<br>Electrical equipment, appliance, and component manufacturing
Paper manufacturing	Paper manufacturing
Explosives manufacturing	Explosives manufacturing
Miscellaneous manufacturing	Miscellaneous manufacturing
Textiles, apparel, and leather manufacturing	Textiles, apparel, and leather manufacturing

50
51

Table S6. Illustrative minimum estimates of chemical-related carbon footprints, production volumes, and total greenhouse gas emissions of organic chemicals used as inputs across 13 industrial sectors in the United States, based on the Chemical Data Reporting. Note that our results represent a minimum estimate of chemical carbon footprints in these sectors, and comparisons between individual sectors should not be done, due to unavoidable data gaps.

Industrial sectors	Num. of chemicals	Percentage of high-carbon footprint chemicals (%)	Amount (tonnes)	Amount share of high-carbon footprint chemicals (w%)	Total chemical-related carbon emissions (kg CO_2-eq)	Overall carbon emission shares of high-carbon footprint chemicals (%)
Petrochemical manufacturing	291	1%	2.62×10^{7}	<1%	4.01×10^{10}	<1%
Construction	186	5%	3.56×10^{4}	4%	1.35×10^{8}	17%
Paper manufacturing	145	14%	8.64×10^{4}	5%	3.84×10^{8}	20%
Adhesive manufacturing	255	9%	3.48×10^{4}	1%	1.28×10^{8}	2%
Pigment and coating manufacturing	1031	11%	7.25×10^{4}	2%	2.47×10^{8}	8%
Food product manufacturing	284	13%	6.78×10^{4}	<1%	1.38×10^{8}	<1%
Textiles, apparel, and leather manufacturing	248	8%	1.99×10^{4}	2%	7.41×10^{7}	9%
Electronic product manufacturing	164	3%	2.09×10^{4}	<1%	9.11×10^{7}	<1%
Cleaning chemical manufacturing	684	9%	2.94×10^{5}	<1%	1.32×10^{9}	2%
Miscellaneous manufacturing	255	4%	2.02×10^{5}	<1%	2.62×10^{8}	<1%
Plastics and rubber manufacturing	1949	21%	7.00×10^{6}	1%	1.99×10^{10}	6%
Agricultural chemical manufacturing	362	8%	6.61×10^{5}	<1%	9.84×10^{8}	4%
Others	3208	10%	5.53×10^{7}	<1%	8.01×10^{10}	1%

Table S7. Molecular frameworks likely associated with a high carbon footprint (>10 kg CO_2-eq/kg).

SMILES	Diagram	Ratio of high carbon footprint chemicals	Frequency in the data set
CC(C1CCCCC1)C1CCCCC1	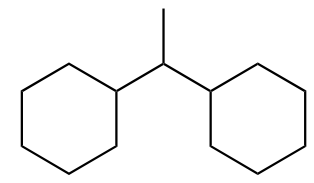	80%	596
CC1C2CCCCC2C(C)C2CCCCC12	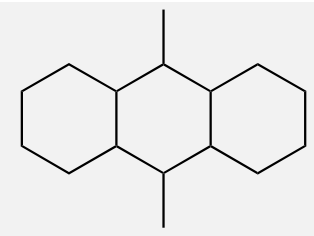	100%	473
C1CCC2CC3CCCCC3CC2C1	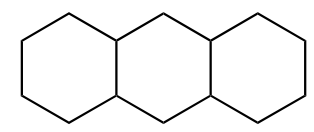	98%	424
C1CCC(C(C2CCCCC2)C2CCCCC2)CC1	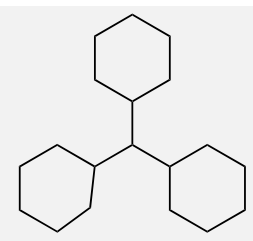	98%	417
C1CCC2C(C1)CC1CCCCC12	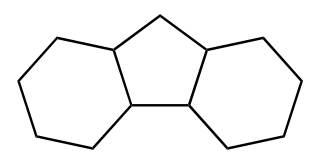	99%	355
C1CCC2C(C1)CCC1CCCCC12	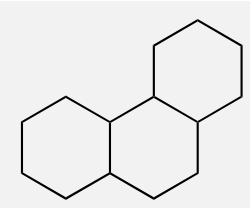	98%	209
C1CC2C3CCC(C3)C2C1	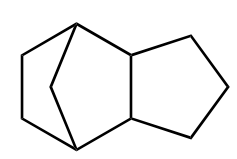	81%	177
C1CCC(C2CC3CCCCC3C2)CC1	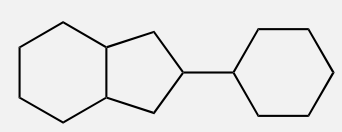	100%	155
C1CCC(CCC2CCCC3CCCCC32)CC1	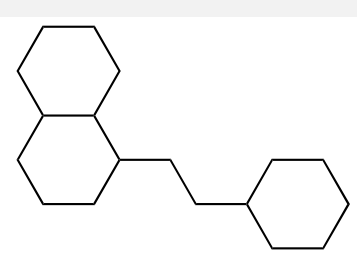	100%	155
CC1CC(C2CCCCC2)CC2CCCCC12	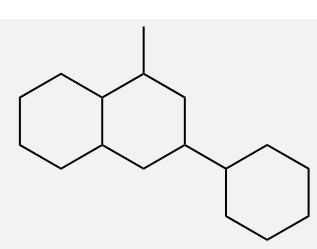	100%	145
C1CCC(C2CCC(C3CCCCC3)CC2)CC1	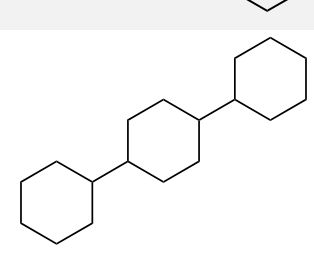	90%	135
C1CCC(CC2CCCC(CC3CCCCC3)C2)CC1	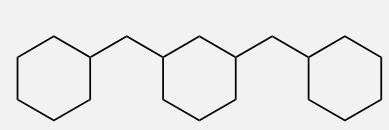	89%	102

Table S8. Functional groups likely associated with a high carbon footprint (>10 kg CO_2-eq/kg).

Functional groups	Description	Diagram	Share of high carbon footprint chemicals	Frequency in the data set
Secondary aromatic amine	A nitrogen bonded to one hydrogen and two organic substituents, one of which is aromatic.	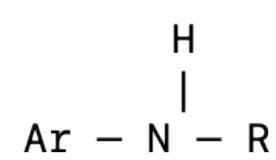	83%	596
Diarylthioether	A sulfur atom bonded to two aryl (aromatic) groups.	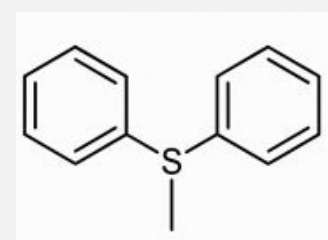	83%	473
Phosphonium	A positively charged phosphorus center bonded to four organic substituents.	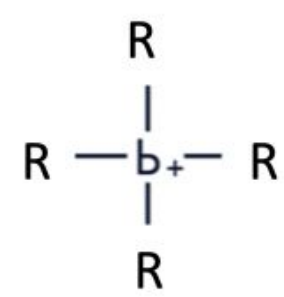	81%	424

References:

1. Australian Department of Health and Aged Care. Australian Inventory of Industrial Chemicals, https://www.industrialchemicals.gov.au/
2. Environment and Climate Change Canada. Domestic Substances List, https://www.canada.ca/en/environment-climate-change/services/canadian-environmental-protection-act-registry/substances-list/domestic.html
3. Health Canada. Canadian Pesticide Product Information Database. https://open.canada.ca/data/en/dataset/e10b0d6e-04ac-4014-a64a-666c3874bbe0
4. ECHA. Pre-registered Substances List. https://echa.europa.eu/information-on-chemicals/pre-registered-substances
5. EU. REACH Regulation. https://environment.ec.europa.eu/topics/chemicals/reach-regulation_en
6. EU. Cosmetic ingredient database. https://single-market-economy.ec.europa.eu/sectors/cosmetics/cosmetic-ingredient-database_en
7. EU. Public Health - Union Register of Medicinal Products. https://ec.europa.eu/health/documents/community-register/html/index_en.htm
8. EU. European Observatory for Nanomaterials. https://euon.echa.europa.eu/
9. National Institute of Technology and Evaluation. Japanese Chemical Substances Control Law. https://www.nite.go.jp/en/chem/kasinn/lists.html
10. National Institute of Technology and Evaluation, Japan. Japanese Industrial Safety and Health Act. https://www.japaneselawtranslation.go.jp/en/laws/view/3440/en
11. Ministry of Natural Resources, Environment and Climate Change, Malaysia. Malaysian Environmentally Hazardous Substances System. https://myehs.doe.gov.my/
12. New Zealand Environmental Protection Authority. New Zealand Inventory of Chemicals. https://www.epa.govt.nz/database-search/new-zealand-inventory-of-chemicals-nzioc/
13. Nordic Council of Ministers Chemical Group. SPIN - Substances in Preparations in Nordic Countries. http://spin2000.net/
14. Philippine Department of Environment and Natural Resources. Philippine Inventory of Chemicals and Chemical Substances. https://chemical.emb.gov.ph/?page_id=138
15. South Korean Ministry of Environment. South Korean Existing Chemicals Inventory. https://www.chemradar.com/inventory-wiki/cpmkemnhys5c

16. Bundesamt für Gesundheit BAG. List of new substances notified or reported in Switzerland. https://www.anmeldestelle.admin.ch/chem/en/home/themen/pflicht-hersteller/stoffe/neuer-stoff.html
17. Occupational Safety and Health Administration, Ministry of Labor, Taiwan. Taiwanese Chemical Substance Inventory. https://www.osha.gov.tw/48783/48784/48851/48853/61086/post
18. United States Environmental Protection Agency. Toxic Substances Control Act Chemical Substance Inventory. https://www.epa.gov/tsca-inventory
19. United States Environmental Protection Agency. Active Pesticide Product Registration Informational Listing. https://ordspub.epa.gov/ords/pesticides/f?p=APPRIL_PUBLIC:2::::::
20. Department of Chemicals - Ministry of Industry and Trade. Vietnam National Chemical Inventory.https://www.chemsafetypro.com/Topics/Vietnam/Vietnam_National_Existing_Chemical_Inventory.html
21. Sun, Y.; Wang, X.; Ren, N.; Liu, Y.; You, S. Improved Machine Learning Models by Data Processing for Predicting Life-Cycle Environmental Impacts of Chemicals. Environmental Science & Technology 2022. DOI: 10.1021/acs.est.2c04945.
22. Song, R.; Keller, A. A.; Suh, S. Rapid Life-Cycle Impact Screening Using Artificial Neural Networks. Environmental Science & Technology 2017, 51 (18), 10777-10785. DOI: 10.1021/acs.est.7b02862.
23. Yap, C. W. PaDEL-descriptor: an open source software to calculate molecular descriptors and fingerprints. Journal of Computational Chemistry 2011, 32 (7), 1466-1474. DOI: 10.1002/jcc.21707.
24. U.S. Environmental Protection Agency. Chemical Data Reporting under the Toxic Substances Control Act. Website https://www.epa.gov/chemical-data-reporting (2024).
25. Logan, H., Astrup, T. F. & Damgaard, A. Additive inclusion in plastic life cycle assessments part I: Review of mechanical recycling studies. Journal of Industrial Ecology 28, 1582-1597 (2024). https://doi.org:10.1111/jiec.13542
26. Logan, H., DeMeester, S., Astrup, T. F. & Damgaard, A. Additive inclusion in plastic life cycle assessments, part II: Review of additive inventory data trends and availability. Journal of Industrial Ecology 28, 1554-1566 (2024). https://doi.org:10.1111/jiec.13534
27. Parvatker, A. G. et al. Cradle-to-Gate Greenhouse Gas Emissions for Twenty Anesthetic Active Pharmaceutical Ingredients Based on Process Scale-Up and Process Design Calculations. ACS

Sustainable Chemistry & Engineering 7, 6580-6591 (2019). https://doi.org:10.1021/acssuschemeng.8b0547
